# From spiral cleavage to bilateral symmetry: the developmental cell lineage of the annelid brain

**DOI:** 10.1186/s12915-019-0705-x

**Published:** 2019-10-22

**Authors:** Pavel Vopalensky, Maria Antonietta Tosches, Kaia Achim, Mette Handberg-Thorsager, Detlev Arendt

**Affiliations:** 1 Developmental Biology Unit, European Molecular Biology Laboratory, Meyerhofstraße 1, 69117 Heidelberg, Germany; 2 Max Planck Institute of Molecular Cell Biology and Genetics, Pfotenhauerstraße 108, Dresden, 01307 Germany

**Keywords:** Cell lineage, Cleavage, Spiralian, Development, Bilateral, Symmetry

## Abstract

**Background:**

During early development, patterns of cell division—embryonic cleavage—accompany the gradual restriction of blastomeres to specific cell fates. In Spiralia, which include annelids, mollusks, and flatworms, “spiral cleavage” produces a highly stereotypic, spiral-like arrangement of blastomeres and swimming trochophore-type larvae with rotational (spiral) symmetry. However, starting at larval stages, spiralian larvae acquire elements of bilateral symmetry, before they metamorphose into fully bilateral juveniles. How this spiral-to-bilateral transition occurs is not known and is especially puzzling for the early differentiating brain and head sensory organs, which emerge directly from the spiral cleavage pattern. Here we present the developmental cell lineage of the *Platynereis* larval episphere.

**Results:**

Live-imaging recordings from the zygote to the mid-trochophore stage (~ 30 hpf) of the larval episphere of the marine annelid *Platynereis dumerilii* reveal highly stereotypical development and an invariant cell lineage of early differentiating cell types. The larval brain and head sensory organs develop from 11 pairs of bilateral founders, each giving rise to identical clones on the right and left body sides. Relating the origin of each bilateral founder pair back to the spiral cleavage pattern, we uncover highly divergent origins: while some founder pairs originate from corresponding cells in the spiralian lineage on each body side, others originate from non-corresponding cells, and yet others derive from a single cell within one quadrant. Integrating lineage and gene expression data for several embryonic and larval stages, we find that the conserved head patterning genes *otx* and *six3* are expressed in bilateral founders representing divergent lineage histories and giving rise to early differentiating cholinergic neurons and head sensory organs, respectively.

**Conclusions:**

We present the complete developmental cell lineage of the *Platynereis* larval episphere, and thus the first comprehensive account of the spiral-to-bilateral transition in a developing spiralian. The bilateral symmetry of the head emerges from pairs of bilateral founders, similar to the trunk; however, the head founders are more numerous and show striking left-right asymmetries in lineage behavior that we relate to differential gene expression.

## Background

During early development, embryonic cleavages produce blastomeres via a rapid series of cell divisions without significant growth, relying on maternally deposited messengers and proteins. During these divisions, the initially broad developmental potential of blastomeres becomes gradually restricted towards distinct cell fates. This can occur via two basic modes: (i) regulative (conditional) development, exhibited by cnidarians, sea urchins, and vertebrates [1], where almost all blastomeres share a broad developmental potential and cell fate determination largely depends on local signaling events, or (ii) mosaic development, where most blastomeres inherit distinct maternal determinants and signaling is assumed to play a minor role. Mosaic development is considered characteristic for the Spiralia, a large group of invertebrate phyla within the clade Lophotrohozoa [2, 3], but also for nematodes [4] and ascidians [5, 6]. It requires differential in ovo localization of determinants, a stereotypic arrangement of cleaving blastomeres and an invariant cell lineage. Recent results, however, hint at a considerable degree of cell-cell signaling also in these species with invariant lineages [7, 8], which underscores that regulative and mosaic development mostly differ in the relative contributions of autonomous versus conditional cell fate determination.

**Fig. 1:**
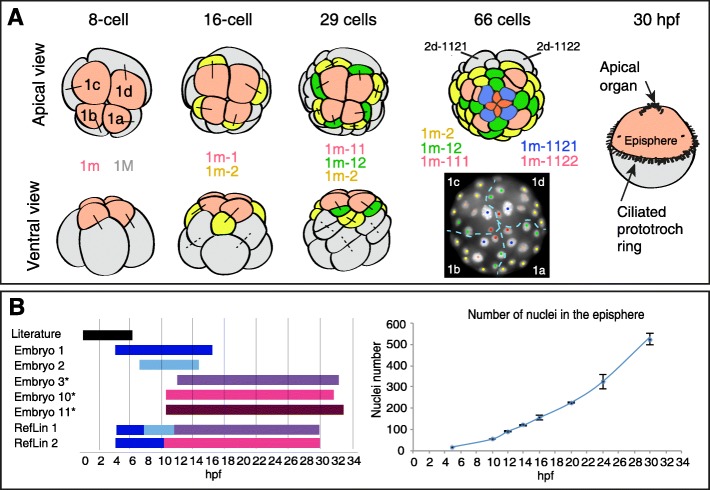
Overview of early spiral cleavage and live imaging of the developing episphere. **a** The early development of a prototroch larva by spiral cleavage. The apical quartet of micromeres 1m (light red) present at 8-cell stage gives rise to the episphere, whereas the quartet of macromeres 1M (gray) gives rise to the hyposphere. The precursors (1m-2) of the prototroch cells are labeled in yellow. For 66-cell stage, a schematic apical view (top) together with a corresponding snapshot (bottom) of the nuclear signal in the time-lapse recording of the developing episphere is shown. The colors of the nuclear tracks in the snapshot correspond to the coloring in the schematic apical view. The dashed blue line represents the border between embryonic quadrants. Apical views modified from [9]. The ventral views are extensively schematized for simplicity. **b** Left-hand panel shows the overview of time-lapse recordings used for the analysis of the cell lineage. Asterisks mark the movies used to create the consensus lineage tree (Additional file 10: Figure S2). The reference lineage movies RefLin1 (Additional file 3 and RefLin2 (Additional file 6) were assembled from 3 or 2 recordings, respectively, schematized by the colored bars along the timeline. To perform temporal calibration of the movie frames to developmental time, total nuclei in the episphere of at least three fixed specimens for each indicated stage (5, 10, 12, 14, 16, 20, 24, and 30 hpf) were counted (plotted in the right-hand panel, error bars represent the standard deviation of the mean, *n* = 3)

In Spiralia, the eponymous “spiral cleavage” produces a highly stereotypic, spiral-like arrangement of blastomeres (Fig. 1a) (reviewed in [10, 11]): The first two cleavages, perpendicular to each other, subdivide the embryo along the animal-vegetal axis into four blastomeres, representing the four future embryonic “quadrants” A, B, C, and D [2]. The subsequent cleavages are asymmetrical, generating quartets of smaller micromeres towards the animal pole and quartets of bigger macromeres towards the vegetal pole. In addition, due to an oblique angle of these divisions, the originating micromere quartets are alternately turned clock- or counter-clockwise against the macromere quartet, so that the micromeres come to be located in the furrows between the macromeres (Fig. 1a). The initial cleavage pattern is identical for each quadrant, so that the whole early embryo shows a fourfold rotational symmetry around the animal-vegetal axis. Corresponding cells with similar lineage in the four quadrants are here referred to as *quadrant homologs*.

In many annelids and mollusks, spiral cleavage produces spherical planctonic larvae called trochophore larvae (Fig. 1a). The larvae form a simple nervous system that integrates sensory information from photo-, mechano-, and chemosensory receptor cells for the control of ciliary locomotion [12–14]. Its most prominent features are an apical nervous system with an apical organ underlying an apical tuft. The apical organ is connected via radial nerves to a ring nerve [10, 11]. The ring nerve innervates a pronounced circular ciliary band, the prototroch, subdividing the larva into an upper episphere and a lower hyposphere. During settlement metamorphosis, the larva transforms into an adult body with overt bilateral symmetry (or more or less complex derivatives thereof, see for instance the development of *Crepidula* [15, 16] and *Ilyanassa* [17]). The former episphere of the larva develops into the head including a prominent pair of cerebral ganglia. The hyposphere gives rise to the trunk including the paired ventral nerve cord [10, 11].

Hence, the most peculiar feature of spiralian development is the transition from the spiral (or rotational) symmetry to an overt bilateral symmetry, which has puzzled embryologists for more than a century (e.g., [18]). How is this spiral-to-bilateral transition accomplished? In the hyposphere, bilateral symmetry is established through the unique behavior of two cells, 2d-112 and 4d [18], that divide once into the left and right bilateral founder cells of the entire trunk and give rise to trunk ectoderm and mesoderm, respectively [19–22]. The situation is more complicated in the episphere, where the bilateral symmetry has to emerge from a pre-existing pattern of spirally arranged micromeres. Here, the spiral-to-bilateral transition may involve a “rearrangement” of micromere position via complex cellular movements, or start from selected bilateral founders—thus analogous to the trunk founders. The latter solution was favored by E. B. Wilson [18], who gave an early and detailed account of spiral cleavage in the annelid *Nereis.* After the appearance of the prototroch, he observed a sudden transition from spiral to bilateral cleavage pattern that he attributed to a pair of (yet to be identified) bilateral founders.

Previous studies in *Platynereis* and other spiralians had established the bilateral fate of early micromeres by injection of tracer dyes, yet did not resolve their lineage in cellular resolution [15, 23, 24]. To understand how bilateral symmetry is established, we reconstructed the full developmental cell lineage for the episphere (apical hemisphere) of the marine annelid *Platynereis dumerilii* from the fertilized egg to the swimming trochophore stage at ~ 30 h post fertilization (hpf). In addition, we linked early lineages to gene expression using a cellular resolution gene expression atlas for several embryonic stages (compare to [25]). This resource is extended here to the episphere undergoing spiral-to-bilateral transition. The time-lapse recordings, software tools, and lineage analysis presented here generate an unprecedented resource for spiralian biology available so far only for nematode and tunicate model systems.

Our lineage analysis allows tracking the spiral-to-bilateral transition in cellular detail. As postulated by Wilson, we identify bilateral founder cells; yet, we observe an extensive array of paired bilateral founders distributed over the entire episphere at around 12 hpf. Some of them, located in the lateral episphere represent quadrant homologs, that is, they stem from similar (i.e., corresponding) lineages in their respective quadrants. Others, located more medially, stem from dissimilar lineages in their respective quadrants. Mapping the expression of the conserved bilaterian head patterning genes *otx* and *six3* to the developmental lineage, we find that lateral *otx* expression marks the bilateral founders with similar lineage, whereas medial *six3* marks those of dissimilar lineage. Moreover, we find that while the *otx+* lateral founders show strong proliferation during larval stages and remain mostly undifferentiated at 30 hpf, the *six3+* medial founders differentiate earlier and give rise, among others, to bilateral pairs of cholinergic neurons in the larval brain. Finally, we find that the apical organ proper does not develop from bilateral founders, but originates from the most medial cells that lack bilateral symmetry.

We relate our findings to the fast succession of two fundamentally distinct phases of spiralian development in *Platynereis*: an early embryonic phase with mosaic and determinate elements, giving rise to rotationally symmetrical cell types of the larval body such as the ciliated prototroch, and a later regulative phase characterized by positional specification of the bilateral founders on each side of the developing head. These two phases appear universally present in the spiralian life cycle.

## Results

### Live imaging and tracking of the cell lineage in the *Platynereis* episphere

The annelid head (the brain and associated sensory organs) is almost entirely formed by the offspring of the apical micromeres 1a, 1b, 1c, and 1d, here collectively referred to as “1m” [23, 26]. The development of the 1m micromeres is easily accessible to live imaging by standard laser scanning confocal microscopy. To track cell divisions in the developing head, we injected embryos at different stages post fertilization (1, 2, or 4 cell stage) with *h2a-rfp* and *lyn-egfp* mRNAs [27], which label chromatin and cell membranes, respectively. Then we recorded time-lapse movies of these apically mounted embryos (Fig. 1a, b, and 4D recordings of each embryo available in online data repository [28]). To track and reconstruct the lineage, we developed a package of simple macros for ImageJ/FIJI [29] allowing manual tracking and visualization of lineage-related information from confocal microscopy stacks (Additional files 1 and 2). We tracked all cell divisions in the episphere of multiple embryos spanning the developmental time from 16-cell stage (~ 2 hpf) until ~ 32 hpf when more than 500 cells are present in the episphere (Fig. 1b), with at least three embryos coverage per developmental stage (original 4D recordings of each embryo available in online data repository [28]), Z-projections combined in reference lineage movies are provided in Additional files 3, 4, 5, 6, 7, and 8. This comprehensive dataset allowed us to perform detailed cell lineage analyses of developmental stereotypicality, clonal behavior, and the transition from spiral to bilateral symmetry.

### The cell divisions follow a stereotypical pattern in the *Platynereis* episphere until swimming larval stages

**Fig. 2:**
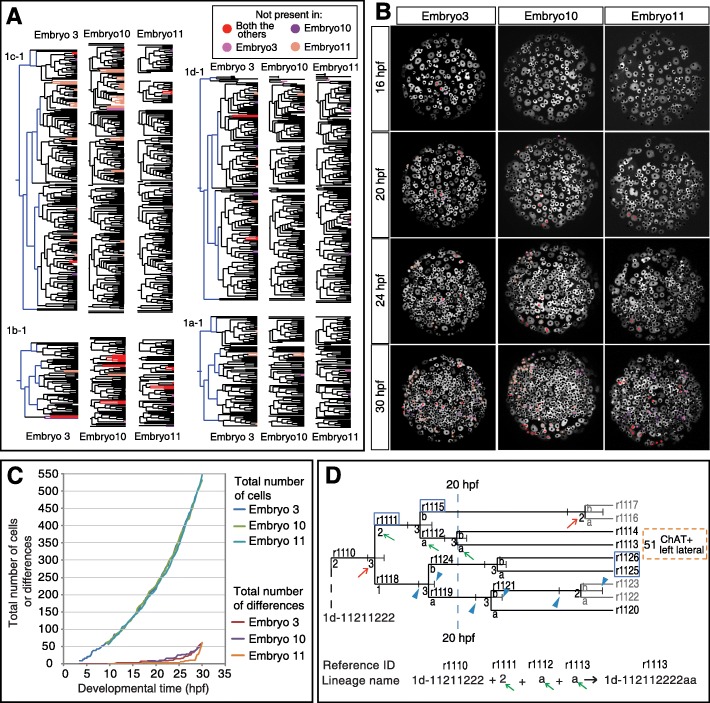
The stereotypicality of episphere development. **a** The comparison of the cell lineage trees of three larvae at 30 hpf. Blue branches in the lineage trees of embryo 3 represent the early developmental lineage (0–6 hpf) described in previous study [23], but not captured by the live-imaging movies in this work. Corresponding cells/divisions conserved in all three larvae are colored in black. The divisions and cells that do not occur in all three larvae are color-coded according to the legend. **b** The snapshots of the Z-projection of the live-imaging movies showing the differences between the three larvae at different time points. Differences are color-coded as in panel **a**. **c** Quantification of differences among embryos. The total number of differences represents the number of cells that are not present in the other two embryos at a given time point. The differences start to appear around 16 hpf and increase with time, reaching around 10% of the total cell number in the episphere at 30 hpf. **d** A cut-out from the consensus lineage tree (Additional file 10: Figure S2) illustrating the annotation system: The reference IDs (examples in blue boxes) are unique identifiers of each cell throughout the lineage tree and track files. Each division produces two daughter cells, whose lineage name is extended by a “1” (or “a”) or “2” (or “b”). The letter of extension is given at the edge connecting the vertical line (representing the division point) and the horizontal branch (green arrows). Using this system, the full lineage name can be read out from the consensus lineage tree. The red arrow at the division point indicates the number of embryos in which the given division occurred (max. 3). If the division occurred in only 2 out of 3 embryos, the subsequent branch lines are shaded in gray, instead of black color

To investigate the reproducibility of cell division patterns across individuals, we injected nuclear tracers into 2- and 4-cell stage embryos and compared the resulting clonal domains with the results of the live imaging at 32 hpf. The clonal domains originating from tracer dye injections were in a good agreement with the shape and position of the clonal domains inferred from the tracked time-lapse movies (Additional file 9: Figure S1A-D’), pointing to a high level of stereotypicality. In addition, the shape and overall arrangement of the clonal domains originating from ~ 13 hpf are highly similar between embryos (Additional file 9: Figure S1E). To address the stereotypicality of episphere development beyond this time point, we identified corresponding cells in different imaged specimens on the basis of lineage information, relative cell positions at division, and cell cycle length (Additional file 9: Figure S1F-H, see the “Materials and methods” section for more details). We compared the time-lapse movies of more than three independent (injections at different days) specimens up to 24 hpf and three specimens until 30 hpf (Fig. 1b). The embryos showed no differences until 16 hpf. Afterwards, the embryos showed a largely stereotypical development, both at the level of the lineage tree topology as well as cell positions, with only a small number of differences distributed over the developing episphere (Fig. 2a–c). These differences could be attributed either to biological variance or to minor late developmental aberrations due to cumulative phototoxicity. The only exception is the ventral apical rosette cell 1b-111, which shows most variability in timing and division pattern observed across larvae (see below). Based on the analyzed embryos and available literature, we generated a consensus lineage tree of the episphere from the egg until 30 hpf and annotated the identified cell types (Fig. 2d and Additional files 10: Figure S2 and 11: Table S1). Taken together, our comparative analysis shows that *Platynereis* brain development is highly stereotypical at the level of overall cell arrangement and lineage tree topology.

### Early differentiating cells show an invariant cell lineage

**Fig. 3:**
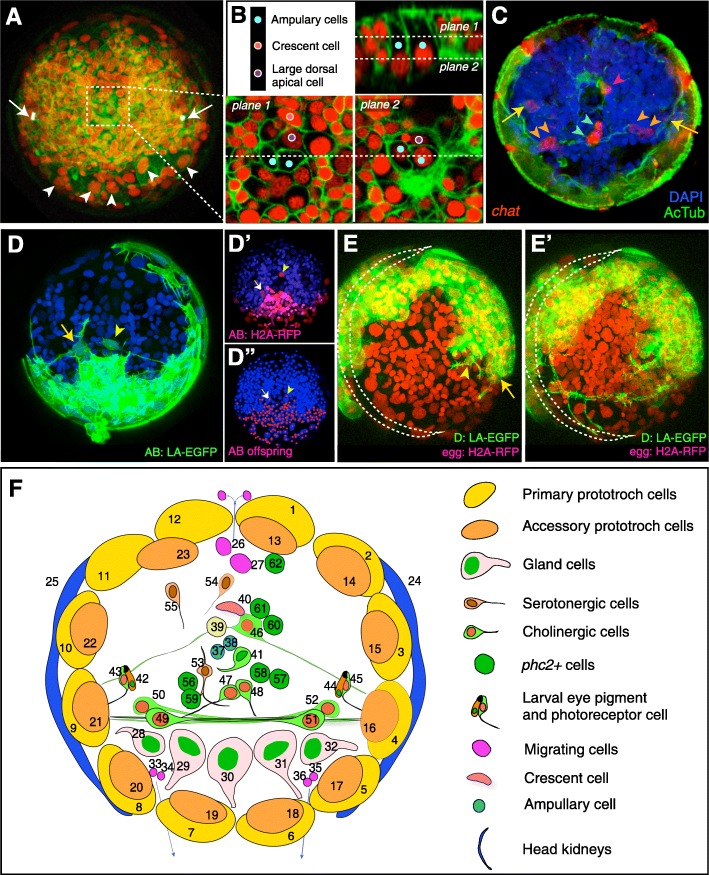
Differentiated cell types with known lineage at 30 hpf. **A**, **B** Cell types identified directly in the last frame of the time-lapse recordings by their position and morphological features. The larval eye pigment cells identified by their autofluorescence in the red spectrum (**A**, white arrows). The large gland cells with the typical flask shape and large size (**A**, white arrowheads). **B** Several cell types of the apical organ could be identified by their morphology and position (described in detail in [14]). **C** The WMISH of *chat* performed on the live-imaged larva (shown in panel **A**) fixed immediately after imaging allows addressing the cell lineages of cholinergic neurons. Yellow arrows indicate the position of larval eye photoreceptors, orange arrowheads the ventrolateral ChAT+ cells, red arrowhead the apical ChAT+ cells with first lateral axons, and blue arrowheads the ventromedial cholinergic cells. **D** Two apical neurons with axons revealed by injection of the AB blastomere with *la-egfp* mRNA. The neuron with the ventrolateral projections lies outside from the rest of the AB-labeled domain (**D**’ and **D**”). **E**, **E**’ The snapshots of a time-lapse recording of larvae injected with *h2a-rfp*mRNA at 1-cell stage and *la-egfp* to the D blastomere shows the axon of a flask-shaped cell in the apical organ (yellow arrowhead) and the growing axon of the ventral cholinergic cells (yellow arrow). The prototroch ring is indicated by a dashed crescent. **F** A summary diagram of the differentiated cell types in the episphere at ~ 30 hpf. The numbering corresponds to the first column of Table 1

To date, the only differentiated cells for which the cell lineage has been fully described in *Platynereis* episphere are the primary prototroch cells [26]. In our time-lapse recordings, several differentiated cell types could be directly identified based on their morphology and other microscopical features: the prototroch cells, the larval eye pigment cells, the five ventral gland cells (Fig. 3A), and several cell types in the apical organ (Fig. 3B) [14]. In addition to morphological and anatomical identification, we also mapped the expression of the cholinergic marker *Choline Acetyl Transferase* (*chat*) on the lineage, by performing whole-mount mRNA in situ hybridization (WMISH) on live-imaged and lineage-tracked embryos fixed just after the last timeframe of the recording (Fig. 3C). At 30 hpf, the *chat* expression pattern comprises nine differentiated cells, mostly involved in controlling cilio-motor behavior [12, 13, 30]. Another hallmark of differentiating neurons is the formation of axons. Zygotic injection of the nuclear marker *h2a-rfp* mRNA followed by the injection of *lifeAct-EGFP* mRNA (labeling actin filaments) into a single blastomere at 2-cell or 4-cell stage allows following the lineage of cells forming axonal projections (Fig. 3D–E’). With this approach, we identified two apical cells projecting outside the AB domain (Fig. 3D–D”) and cells with axons traversing the dorso-ventral midline (Fig. 3E, E’). We also observed several apoptotic cells characterized by condensation and later dissociation of nuclear content, showing the same lineage in all investigated embryos (Fig. 3F). In addition, to link cellular lineages to differential gene expression, we conducted WMISH expression analysis for markers for cholinergic neurons—*choline acetyltransferase* (*chat*), for neuropeptidergic neurons—p*rohormone convertase 2* (*phc2*), and for glutamatergic neurons—*vesicular glutamate transporter* (*vglut*) (Fig. 3F and Additional file 12: Figure S3). In total, we addressed the cell lineages of 62 non-dividing, presumably differentiated cell types in the 30 hpf episphere, summarized in (Fig. 3F and Table 1).

**Table 1:** The cell lineage of the differentiated cell types at 30 hpf

No.	Cell type	Lineage	Support	Last observed division	rID
1	Posterior prototroch 1	1d-221	Refs. + (2/2)	~ 7 hpf	r1365
2	Posterior prototroch 2	1d-222	Refs. + (2/2)	~ 7 hpf	r1366
3	Posterior prototroch 3	1d-212	Refs. + (2/2)	~ 6.5 hpf	r1363
4	Posterior prototroch 4	1a-221	Refs. + (2/2)	~ 7 hpf	r215
5	Posterior prototroch 5	1a-222	Refs. + (2/2)	~ 7 hpf	r216
6	Posterior prototroch 6	1a-212	Refs. + (2/2)	~ 7 hpf	r213
7	Posterior prototroch 7	1b-221	Refs. + (2/2)	~ 7 hpf	r361
8	Posterior prototroch 8	1b-222	Refs. + (2/2)	~ 7 hpf	r362
9	Posterior prototroch 9	1b-212	Refs. + (2/2)	~ 7 hpf	r359
10	Posterior prototroch 10	1c-221	Refs. + (2/2)	~ 7 hpf	r899
11	Posterior prototroch 11	1c-222	Refs. + (2/2)	~ 7 hpf	r900
12	Posterior prototroch 12	1c-212	Refs. + (2/2)	~ 7 hpf	r897
13	Anterior prototroch 1	1d-1221	(3/3)	~ 8.5 hpf	r1356
14	Anterior prototroch 2	1d-211	Refs. + (2/2)	~ 6.5 hpf	r1362
15	Anterior prototroch 3	1a-1212	(3/3)	~ 8.5 hpf	r208
16	Anterior prototroch 4	1a-122	(3/3)	~ 7 hpf	r209
17	Anterior prototroch 5	1a-211	Refs. + (2/2)	~ 7 hpf	r212
18	Anterior prototroch 6	1b-1212	(3/3)	~ 8.5 hpf	r354
19	Anterior prototroch 7	1b-122	(3/3)	~ 7 hpf	r355
20	Anterior prototroch 8	1b-211	Refs. + (2/2)	~ 7 hpf	r358
21	Anterior prototroch 9	1c-1212	(3/3)	~ 8.5 hpf	r892
22	Anterior prototroch 10	1c-122	(3/3)	~ 7 hpf	r893
23	Anterior prototroch 11–12	1c-211	Refs. + (2/2)	~ 7 hpf	r896
24	Left head kidney	1d-11,221	(5/5)	~ 7 hpf	r1133
25	Right head kidney	1c-11221	(5/5)	~ 7 hpf	r613
26	Dorsally migrating 1^st^ wave	1d-1222	(5/5)	~ 8.5 hpf	r1357
27	Dorsally migrating 2^nd^ wave	1d-121	(5/5)	~ 7 hpf	r1352
28	Gland most right (GRR)	1b-112211121	(3/4)	~ 15.5 hpf	r267
29	Gland middle right (GMR)	1b-11221222	(3/3)	~ 13.5 hpf	r295
30	Gland middle (GMM)	1b-121122	(3/3)	~ 13 hpf	r353
31	Gland middle left (GML)	1a-11221222	(3/3)	~ 13.5 hpf	r126
32	Gland most left (GLL)	1a-112211121	(3/3)	~ 15.5 hpf	r118
33	Apoptotic upper right	1c-12111	(3/3)	~ 10.5 hpf	r838
34	Apoptotic lower right	1b-1122112	(3/3)	~ 11 hpf	r290
35	Apoptotic upper left	1a-12111	(3/3)	~ 10.5 hpf	r158
36	Apoptotic lower left	1a-1122112	(3/3)	~ 11 hpf	r122
37	Ampullary cell right	1c-1112	(3/3)	~ 9 hpf	r367
38	Ampullary cell left	1c-1111	(3/3)	~ 9 hpf	r366
39	Large dorsal apical cell	1d-1112	(5/5)	~ 10 hpf	r905
40	Crescent cell	1c-112121222	(4/4)	~ 17.5 hpf	r574
41	First axon apical organ	1d-1111	(3/3)	~ 10 hpf	r904
42	Larval eye pigment cell right	1c-121121b	(3/3)	~ 16 hpf	r842
43	Larval eye photoreceptor right, *chat*+	1c-121121a	(3/3)	~ 16 hpf	r841
44	Larval eye pigment cell left	1a-1211211	(3/3)	~ 16 hpf	r206
45	Larval eye photoreceptor left, *chat*+	1a-1211212	(3/3)	~ 16 hpf	r207
46	*chat+* apical dorsal, *phc*2+, first bilateral axons	1a-11111	(3/3)	~ 11.5 hpf	r5
47	*chat+* apical ventral peripheral (AVP)	1a-11212112b	(3/3)	~ 16.5 hpf	r86
48	*chat+* apical ventral medial (AVM)	1a-11211212b	(3/3)	~ 18 hpf	r50
49	*chat+* right lateral bigger (RLP)	1c-112112222ba	(4/5)	~ 20 hpf	r525
50	*chat+* right lateral ventral (RLV)	1b-11211221	(3/4)	~ 16.5 hpf	r241
51	*chat+* left lateral bigger (LLP)	1d-112112222aa	(3/3)	~ 20 hpf	r1113
52	*chat+* left lateral ventral (LLV)	1d-112112221nn	(2/2)	~ 28/~ 29 hpf	r1125/r1121
53	The right apical cell with axon, *5HT+, phc2+*	1b-1121111	(1/1)	~ 14.8 hpf	r226
54	The most dorsal serotonergic, *5HT+*	1c-112122122a	(1/1)	~ 17.5 hpf	r583
55	Asymmetricserotonergic, *5HT+*	1c-112222211bb	(1/1)	~ 22 hpf	r834
56	*phc2+* close to the right apical cell with axon, more dorsal	1b-1121112	(1/1)	~ 14.8 hpf	r227
57	*phc2+*	1a-1122121	(1/1)	~ 11.5 hpf	r124
58	*phc2+*	1a-11112b	(1/1)	~ 15.6 hpf	r24
59	*phc2+*, close to the right apical cell with axon, more ventral	1b-1122121	(1/1)	~ 11.5 hpf	r292
60	*phc2+* close to the crescent cell, more ventral	1d-112121	(1/1)	~ 10 hpf	r1128
61	*phc2+* close to the crescent cell, more dorsal	1d-1121221	(1/1)	~ 15.2 hpf	r1130
62	Dorsal medial *phc2+*	1d-1121222	(1/1)	~ 15.2 hpf	r1131

The numbers in the first column correspond to the numbering in Fig. 3F. The column “Lineage” contains the consensus lineage name based on the literature and multiple time-lapse recordings. The “Support” column indicates the number of time-lapse recordings with the given cell lineage/the number of total time-lapse recordings analyzed for a given cell type. “Refs” indicates additional support from published literature [23]. The column “Last observed division” refers to the time point at which the last cell division was observed. Since most of the cells in this table show terminal differentiation characteristics, we consider the time of the last observed division being identical to cell cycle exit. The “rID” column contains the reference ID of the given cell type which corresponds to the rID in the tracking files (Additional files 4 and 7 and the tracking files of individual embryos provided in online data repository [28]) and the consensus lineage tree (Additional file 10: Figure S2)

Because the stereotypic tree topology and cell positions suggest an invariant cell lineage, the same cell types should be produced by the same cell lineage in different embryos. Indeed, for the vast majority of cell types with the last cell division observed before ~ 15 hpf, the cell lineage is strictly conserved among multiple embryos (column “Support” in Table 1). Interestingly, the cell lineage varies in later-born cells, e.g., *chat+* cell r1125/r1121 (no. 52 in Table 1) exiting the cell cycle at ~ 28 hpf and cell r525 (no. 49 in Table 1) exiting at ~ 20 hpf. In summary, our analyses show that the *Platynereis* larval brain develops via stereotypical cell divisions and that the lineage of early differentiating neuronal cell types is highly reproducible between specimens.

### A gene expression atlas for embryonic and early larval stages

Next, we linked cellular lineages to gene expression, to gain insights into the potential role of apical transcription factors and the identity of differentiating cell types. For this, we generated a whole-mount in situ hybridization (WMISH) atlas with a total of 23 genes for 7 stages (12, 14, 16, 20, 24, 30, and 34 hpf) (Additional files 12: Figure S3 and 13: Table S2). Since most of the identified early differentiating larval cells represent neural cell types, we included neural regionalization and specification transcription factors from the homeodomain, basic helix-loop-helix (bHLH) and zinc finger family (Additional file 12: Figure S3A-B) and general neural differentiation markers (Additional file 12: Figure S3C).

Using our collection, we found that the transcription factors *coe*, *ngn*, *neuroD*, and *prox* are co-expressed with the neuronal differentiation markers *elav and syt*, the cholinergic marker *chat*, and the neuropeptidergic marker *phc2* in the apical organ cells (no. 46 and no. 53, later serotonergic, in Table 1). At later stages, even when expressing cells could no longer be identified individually, our analysis revealed expression correlations and transcriptional dynamics in neural lineages. For example, the expression of the neuronal specification factors *prox*, *ngn*, and *neuroD* appears to always faithfully anticipate expression of the pan-neuronal marker *elav* (compare Additional file 12: Figure S3A-C)*.* Similarly, we observed that expression of the bHLH factor *coe* precedes the expression of cholinergic markers *vacht* and *chat* several hours later (compare Additional file 12: Figure S3, panels A and C), in line with the evolutionary conserved role of COE factors in specification of cholinergic neurons [31]. Interestingly, the expression of the two neuronal differentiation markers *phc2* and *syt* remains restricted to the apical organ region between 24 and 34 hpf, partially overlapping with the cholinergic markers *chat* and *vacht*. This suggests that cholinergic and neurosecretory cells form the core of the larval apical nervous system, in line with single-cell RNA sequencing results [32]. The restricted and stable expression of *phc2* and the cholinergic markers contrasts with the rather dynamic expression of *neuroD*, *ngn*, and *elav* that demarcate neuronal specification more broadly in the developing cerebral ganglia*.*

### Lineages not transitioning to bilateral symmetry

**Fig. 4:**
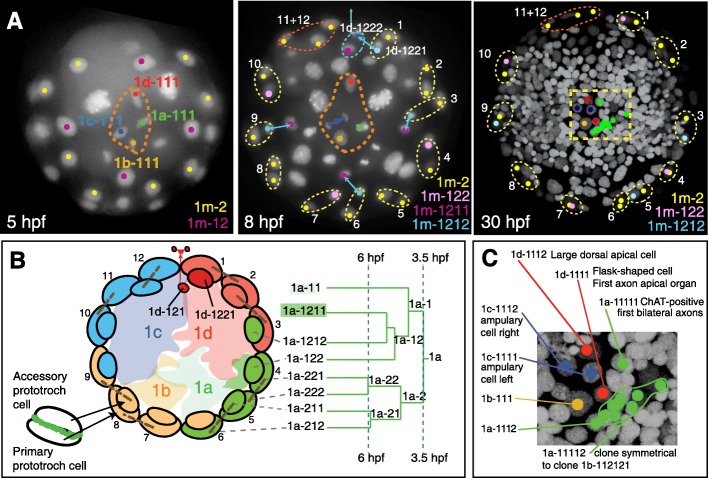
The developmental origin of the accessory prototroch cells and the cells in the apical organ. **a** The overview of the development of the apical rosette (1m-111, orange dashed line) and the primary (1m-2, yellow) and accessory (1m-12, pink) prototroch cells at 5, 8, and 30 hpf. **b** Schematic diagram showing the lineage origin of the prototroch cells. Only the 1a quadrant lineage tree is shown here for simplicity. The cells in the scheme are color-coded by their quadrant of origin. The black dashed lines indicate sister cells. Each quadrant contributes three primary prototroch cells (1m-221, 1m-222, 1m-212) and three accessory prototroch cells (1m-211—a sister cell of one of the primary prototroch cells, 1m-1212 and 1m-122). The only exception is the 1d quadrant producing only two accessory prototroch cells, due to the migration of the 1d-121 clone out of the episphere (see main text for details). Note that the triplet of the accessory prototroch cells originating from a given quadrant does not associate with the triplet of the primary prototroch cells of the same quadrant, but is rotated by one cell counter-clockwise. **c** A detailed scheme of the lineage origin of the apical organ cells

The *Platynereis* cell lineage tree and the gene expression atlas can be used to analyze the symmetry properties of individual cell lineages, in combination with gene expression, cell type, and cell differentiation. We first focused on lineages that retained the initial rotational symmetry, or gave rise to unpaired, non-bilateral descendants along the axis of symmetry. In *Platynereis*, these lineages give rise to early differentiating cells of the prototroch, apical organ, and apical neurosecretory cells (Fig. 4).

The primary prototroch develops from the two vegetal-most quartets of the first micromeres, that is, 1m-22 and 1m-21, in a strictly radial arrangement (Fig. 4a, b). The blastomeres 1m-12, located slightly more apically, divide twice in a spiral mode (with an exception of 1d-12, see below) (Fig. 4b). They produce the non-dividing accessory prototroch cells 1m-122 and 1m-1212. The primary prototroch cells form a rotationally symmetric, almost closed ring around the larval episphere. This ring forms a barrier between the episphere and hyposphere, only allowing cells to pass through a small “gap” between 1c-212 and 1d-221. We observed that episphere cells from the lineages 1d-12 and 1c-112 migrate down to the hyposphere through this small passage (visible in Figs. 3F and 4b).

**Fig. 5:**
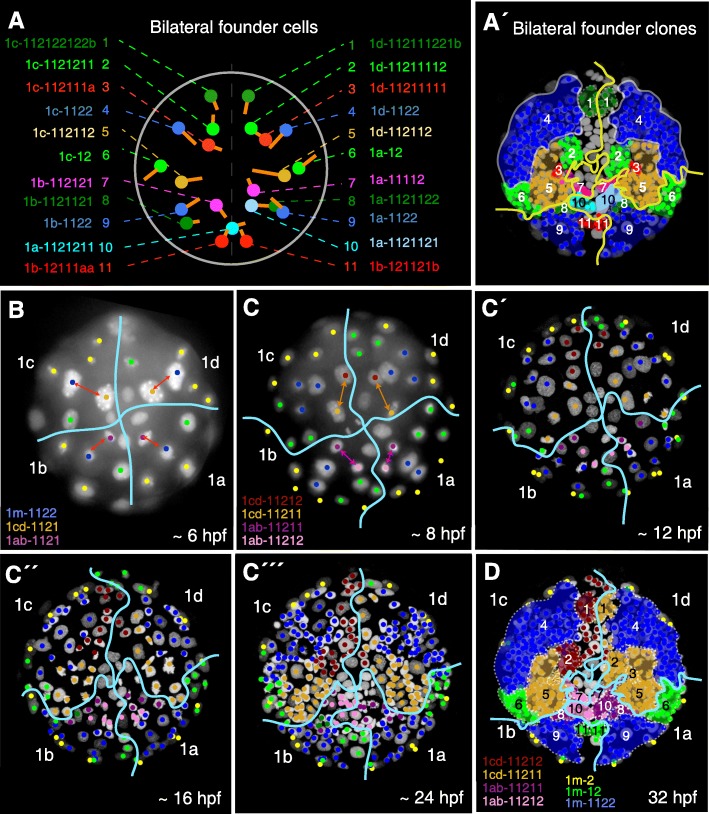
Bilateral founder cells in the episphere. **A** The map of bilateral founder cells summarizes the position of all bilateral founders over time. The orange lines represent the division axis between the bilateral founder (colored spot) and its sister cell. The full lineage name is given for each bilateral founder. **A**’ The clonal offspring of the bilateral founders at 32 hpf, domains numbered according to panel **A**. The tracks with the same coloring is available in Additional files 4 and 7. **B**–**E** The first bilaterally symmetric divisions do not produce bilaterally symmetric clones. The offspring of the early bilaterally dividing cells at 6 hpf (**B**) and 8 hpf (**C**) was highlighted with different colors and their clonal domains were followed until 32 hpf (**C’**–**C”’**, **D**). Note the asymmetrical domains produced by the medial quadrant homologues 1m-11212 and 1m-11211 contrasting with the bilateral domains produced by the lateral quadrant homologues 1m-1122, and the medial region completely devoid of symmetrical clones. See Additional file 14: Figure S4 for more details

The apical organ develops from the four cells 1m-111 that form a prominent “apical rosette” in early development, characteristic for the spiral cleavage pattern [26] (Fig. 4a). These cells produce the early differentiating cells of the apical organ (Fig. 4c) that, together with the prototroch cells, form the first neuromotor circuit. A single division of 1c-111 produces the two ampullary cells described previously [14]. The two daughters of 1d-111 form the “large dorsal apical cell” and one of the flask-shaped cells of the apical organ [33]. The cell 1a-111 buds off the cell 1a-1112 of unknown identity at around 9 hpf. The second daughter cell (1a-1111) divides at around 12 hpf to give rise to the first ChAT-positive cell (1a-11111). Its sister cell (1a-11112) divides multiple times, eventually producing a clone with bilateral symmetry to the clone descendant from 1b-112121 (purple clones in Fig. 5G), providing an example of bilateral clones not related by lineage (see below). The ventral rosette cell 1b-111 shows variable behavior among embryos, from no division (3/6 observed embryos) to one division (2/6 embryos) or more divisions (1/6 embryos). The timing of the first division of 1b-111 ranges from ~ 12 to ~ 24 hpf. The large nuclear volume and rather low nuclear marker signal resemble the highly proliferative blast cells and suggest a possible proliferation in later development.

The apical neurosecretory cells develop from the 1m-112 cells, which mostly give rise to bilateral clones (see below), but also produce a small set of descendants located along the dorsal midline and surrounding the apical organ (Fig. 3F and Table 1). These cells exit their last division before 15 hpf and differentiate as neurosecretory cells expressing the neuropeptidergic marker *phc2* (Table 1).

In summary, our analysis reveals that the prototroch, consisting mostly of cells exiting the cell cycle at ~ 6 hpf, completely retains the rotational symmetry of the spiral cleavage pattern. The medially positioned cells of the apical organ and the medial neurosecretory cells do not show any signs of rotational or bilateral symmetry.

### An array of paired bilateral founder cells

We next determined whether the bilaterally symmetrical *Platynereis* brain and head sensory organs would develop from bilateral founder cells, as do the ventral nerve cord and the trunk mesoderm that develop from the left and right descendants of the 2d-221 and 4d, respectively [18–22]. We defined “bilateral founders” as cells that would (i) have a bilateral counterpart (in position), (ii) produce bilaterally symmetrical clonal progeny with similar lineage tree topology, and (iii) appear at roughly the same developmental time point. Following this definition, we identified not only few, but a whole array of 11 pairs of bilateral founders situated on the right and left sides of the *Platynereis* episphere (Fig. 5A). These appear in succession, starting as early as 6 hpf and continuing to arise until 18 hpf (Additional file 14: Figure S4). These bilateral founders produce clonal progeny that covers large part of the episphere at 32 hpf (Fig. 5A, A’ and Additional files 4 and 7).

Using our tracked lineage, we then determined how these 11 pairs of bilateral founders relate back to the lineage of the spiral cleavage pattern. Previous reports on *Nereis* [18] and *Platynereis* [26, 34] identified the first divisions with bilateral symmetry starting from 7 hpf, yet could not track the progeny of these cells at subsequent stages. Using our tracked lineage, we identified the first “bilateral” divisions (i.e., divisions with a bilaterally rather than rotationally symmetrical orientation of spindle poles) and determined their clonal progeny. Succeeding the fourth spiral cleavage, the 1m-112 cells are the first to divide bilaterally around 6 hpf, producing two bilaterally positioned daughter cells (1m-1121 and 1m-1122) (Fig. 5B). Of these, the more peripherally located cells 1m-1122 (blue in Fig. 5B) represent the first bilateral founder pairs (4 and 9). Around 8 hpf, the more medial 1m-1121 cells divide again in a bilaterally symmetrical manner (Fig. 5C; with lineage homologs of C/D, and A/B quadrants shown in similar color). This results in 4 pairs of bilaterally arranged micromeres (1cd-11211; 1cd-11212; 1ab-11211; 1ab-11212). Of these, the two dorsal pairs (1cd-11211; 1cd-11212) give rise to the bilateral founder pairs 1, 2, 3, and 5 (Fig. 5A), whereas the two ventral pairs (1ab-11211; 1ab-11212) give rise to bilateral founder pairs 7, 8, and 10 (Fig. 5D, with quadrant homologs shown in similar color). Unexpectedly, however, this occurs in a highly asymmetric fashion: Both the dorsal and the ventral pairs proliferate differentially and expand into different episphere territories (Fig. 5C–C”’), so that bilateral founders 1, 2, 3, 7, 8, and 10 arise from non-corresponding lineages (compare to Fig. 5A).

Our analysis thus revealed that the transition from rotational to bilateral symmetry involved very different strategies for different bilateral founder clones: The bilateral founders located more laterally (blue domains 4 and 9 in Fig. 5A’) show an equivalent lineage history between right and left quadrants, whereas the bilateral founders located more medially (domains 1, 2, 3, 7, 8, 10, and 11 in Fig. 5A’) arise from non-equivalent lineages (Fig. 5A’, D and Additional file 14: Figure S4).

Finally, we noted a peculiar difference in how the four initial quadrants 1a, 1b, 1c, and 1d contributed to the multiple pairs of bilateral founders. Overall, the cell clones originating within the 1c quadrant are bilaterally symmetrical to the clones of the 1d quadrant, and the clones originating in the 1b quadrant are symmetrical to the clones of the 1a quadrant. In a few rare cases, however, pairs of bilateral founders came from the 1a versus 1c quadrants (lateral light green clone no. 6 in Fig. 5A’ and D), or originated from a single quadrant (light and dark blue clones in Additional file 14: Figure S4C and dark green clones in Additional file 14: Figure S4D). These results demonstrate that the overall bilaterally symmetric *Platynereis* episphere at 32 hpf originates as a patchwork of different clonal domains showing spiral, bilateral, and no symmetry.

### Early *six3* and *otx* expression matches spiral lineage quartets

**Fig. 6:**
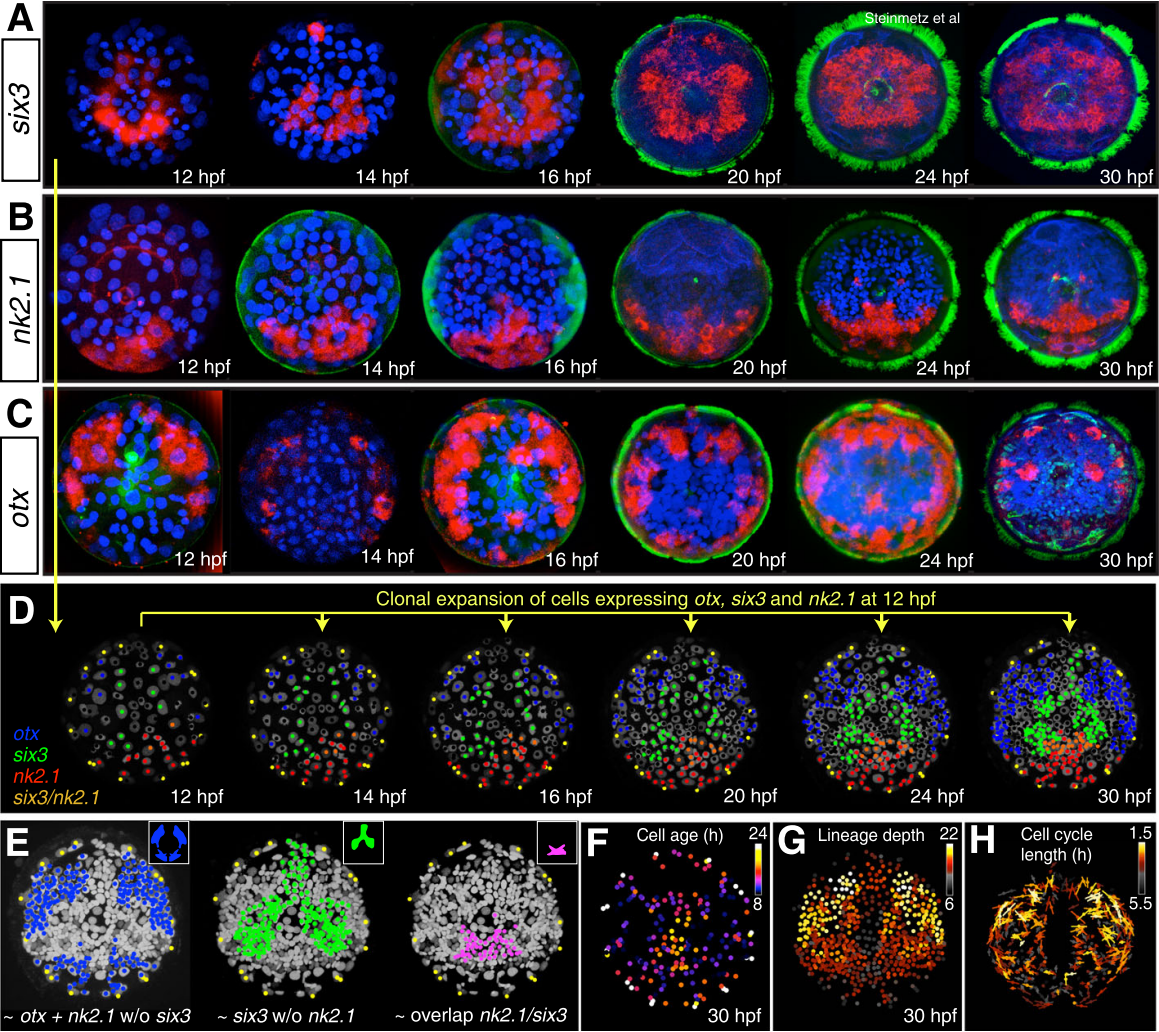
Developmental expression patterns of ancestral early patterning genes. **a**–**c** Representative developmental expression of *six3* (**a**), *nk2.1* (**b**), and *otx* (**c**) between 12 and 30 hpf. **d** The expression of the three genes *otx* (blue), *six3* (green), and *nk2.1* (red) mapped on the lineage movie at 12 hpf and the clonal offspring of these cells visualized at later stages. Cells expressing both *six3* and *nk2.1* are labeled in orange. **e** Whereas the *otx* and *nk2.1* clonal domain reflects the lateral regions adopting bilateral symmetry very early (compare to Figs. 5A' and 7D), the *six3* domain encompasses the medial region with later and lineage-unrelated origin of bilateral symmetry (compare to Fig. 7C and D). **f** Visualization of cell age (time from the last division) at 30 hpf reveals the prototroch and the apical organ as the earliest differentiating regions of the episphere (compare to expression of the neural markers in Additional file 12: Figure S3). **g** Analysis of the lineage depth (the number of preceding cell divisions of a given cell, starting from the zygote until the given time point) identifies the lateral regions as the most proliferative in accordance to the shortest cell cycle length (**h**)

A number of recent studies have revealed a conserved role of the homeodomain transcription factors *six3*, *otx*, and *nk2.1* in the specification of the apical region [14, 33, 35]. In general, a ring of *six3* expression occurs most apically, surrounded by another ring of *otx* expression. *Nk2.1* is expressed in the ventral apical region, overlapping partially with *six3* and *otx*. Taking advantage of our cellular atlas, we set out to characterize the developmental lineage of the *six3*, *otx*, and *nk2.1-*expressing cells. At 6 hpf, *otx* is expressed in the 1m-12 primary trochoblast cells (Additional file 15: Figure S5), which later give rise to the accessory prototroch. At 12 hpf, the cells expressing *otx* match the 1m-1122 descendants with few exceptions (Fig. 6c, e), thus including the bilateral founders that produce the set of bilateral clones with equivalent lineages (representing quadrant homologs, compare Fig. 5D). This means, the early *otx* domain develops from specific quartets of micromeres, which is in line with a possible specification by maternal determinants.

In contrast, at 12 hpf *six3* expression matches the 1m-1121 quartet (Figs. 6a and 5B), which produces bilateral founders with non-equivalent lineages (compare Fig. 5D). Just like the early *otx* domain, this would allow the early *six3* domain to be set up by maternal determinants inherited by the respective quartet. However, in contrast to the *otx+* domain, the bilateral founders emanating from *six3+* domain do not represent quadrant homologs and are thus unlikely to be specified maternally.

The 12 hpf *nk2.1+* clones are partially co-expressing *otx* and *six3*. The *nk2.1+* clones represent the region with the highest disorder with regard to bilateral founder cells. Notably, the ancestral patterning genes *six3*, *otx*, and *nk2.1* are absent from the early differentiating apical organ cells that stem from the 1m-111 lineages.

### Larval *six3* and *otx* expression matches bilateral clones

We next analyzed and compared the expression domains of *six3*, *otx*, and *nk2.1* at later developmental time points up to 30 hpf (Fig. 6a–d). At these larval stages, the *six3* and *otx* expression domains largely remain mutually exclusive, except for a paired domain of overlap left and right of the apical organ (stars in Fig. 6a and c). However, comparing the later expression domains to the clonal progeny of the early *six3+*, *otx*+, and *nk2.1+* cells, we noted that the later *six3* expression spreads into the *otx* clonal descendants (compare Fig. 6a and d at 24 hpf), while *otx* expression is largely turned off in these cells from 20 hpf onwards. *Nk2.1* expression is less dynamic and largely remains expressed in the clonal descendants of its earlier expression (compare Fig. 6b, d). Therefore, while the complementary nature of the ring-shaped *six3* and *otx* domains persists, they appear to shift across the episphere so that they no longer match quartet descendants.

We noted that at larval stages, the *six3* and *otx* domains more closely matched the outlines of bilateral clones and subclones. For example, at 30 hpf, the ventral stripe of *six3* expression largely covered the bilateral founder clones 5, 8, and 10 (compare Figs. 5F and 6a). In addition, the dorsal patches of *six3* expression appeared to match large lineage subclones of the bilateral founder clones 4 (compare Figs. 5F and 6a; green and bright blue subclones in Additional file 14: Figure S4A). The paired patches of *six3* and *otx* co-expression similarly matched a subclone of the bilateral founder clone 5 (compare Figs. 5F and 6c; light brown subclone in Additional file 14: Figure S4B).

Characterizing the *six3*, *otx*, and *nk2.1* domains further, we noted that the *six3* cells generate several differentiated cells at 22 hpf, including the crescent cell (no. 40 in Table 1 and Fig. 2f), *six3* ventral ChAT+ cells (no. 47, 48, 49, 50, 51, 52 in Table 1) (partially co-expressing *nk2.*1), and one serotonergic cell (no. 53 in Table 1). In line with early differentiation, the *six3* cells divide less on average (compare Fig. 6e, g). In contrast, the dorsal *otx* domain is the most proliferative among episphere cells in that it shows the highest lineage depth and the shortest cell cycle length (Fig. 6g, h). Except for the prototroch and accessory prototroch cells, it produces no differentiated cells until 22 hpf (whereas the ventral cells 1ab-1122 give rise to the gland cells, Table 1). Cells in this territory differentiate much later, such as the adult eyes [36].

## Discussion

We have tracked the full cell lineage of the larval episphere in the marine annelid *Platynereis dumerilii*, from spiral cleavage to fully bilateral larval stages, including individual lineages for 62 differentiated cells. Overall, our data confirm earlier observations that the development of spirally cleaving embryos is highly stereotypic at early stages (up to 6 hpf), and extend the notion of stereotypicality to larval stages. Consistent with this, we find that the cell lineage of early differentiating cells is highly invariant.

To relate the *Platynereis* lineage to gene expression and cell identities, we built a gene expression atlas for embryonic and early larval stages, for 23 genes with known roles in developmental specification and cellular differentiation. This is part of ongoing efforts [25, 32, 37, 38] to resolve and understand *Platynereis* development at single cell level. The comparison of our new resources to similar pioneering efforts in other developmental models (e.g., [39–43]) will be especially rewarding for our understanding of conservation and divergence in gene expression profiles and cell types among spiralians.

### Rotational symmetry of early differentiating larval cells

Our lineage analysis corroborates earlier findings that the early differentiating prototroch cells have a strictly spiral origin, and we further show how the diverse, early-appearing cells of the apical organ each emerge from most apical micromeres, via dissimilar lineages. Earlier work in *Platynereis* [44] and early cell dissociation experiments in *Nereis* [45] pointed to a high degree of cell autonomous differentiation for these cells via the inheritance of maternal determinants, and in line with this, several studies in mollusks [46–48] and in *Platynereis* [49] demonstrated that mRNA segregation into specific blastomeres during the cleavage plays a crucial role in cell autonomous specification.

We further show that the spiral and bilateral division patterns co-exist for a certain period, with the first bilateral divisions beginning at ~ 6 hpf while the last spiral divisions of accessory prototroch cells take place at ~ 8 hpf. In line with the notion that the zygotic expression is necessary for the first bilaterally symmetric division in the leech *Helobdella* [50], we did not observe any bilateral behavior before the onset of zygotic transcription [51].

### Highly complex transition from rotational to bilateral symmetry

**Fig. 7:**
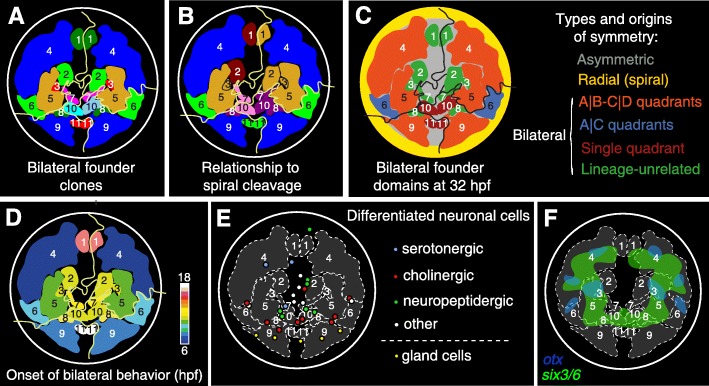
Relationship between bilateral founder domains, cell differentiation, and *otx*-*six3/6* expression. **a** Schematic representation of the bilateral founder domains at 32 hpf with a color code reflecting bilateral symmetry (compare to Fig. 5A’). **b** Schematic representation of the bilateral founder domains color-coded by quadrant homology (compare to Fig. 5D). **c** A summary diagram of the types of symmetry in the episphere. The bilateral founder pairs are color-coded by the type of symmetry behavior (left panel) and their clonal domains at 32 hpf. **d** Bilateral founder cells originate at different time points during development, highlighted by the temporal color-coding of their clonal domains. Note that the peripheral areas (dark and light blue) are the first to adopt the bilateral behavior. The areas along the dorso-ventral axis (yellow, red, and white) are the last ones to adopt bilateral behavior. **e** The position of differentiated cell types (compare to Fig. 3F) within the bilateral founder domains. **f** The overlay of the *otx* and *six3/6* expression and the bilateral founder domains at 32 hpf

Our full lineage analysis until 32 hpf has allowed the first in-depth investigation of the transition from the embryonic spiral cleavage pattern with rotational symmetry to the bilateral symmetry of the early juvenile. As anticipated by Wilson [18], we find that the bilaterally symmetrical parts of the larval body emerge from so-called bilateral founders. However, the generation of these bilateral founders from the four rotational quadrants is surprisingly complicated (Fig. 7a–c). First, the more lateral bilateral founders emerge from equivalent lineages in different quadrants, located on the future left and right body sides. Most of these are located in quadrants A and B (A|B symmetry), or in C and D (C|D symmetry, red regions in Fig. 7c), whereas one bilateral founder pair is shared between A and C (A|C symmetry, blue regions in Fig. 7c). Remarkably, while the A|C bilateral symmetry is less frequent in *Platynereis* and in other annelids such as *Capitella* [24], it has shown to be predominant in the mollusks *Ilyanassa* and *Crepidula* [15, 52]. Second, sets of bilateral founders can emerge from two cells of dissimilar (non-corresponding) lineage in left-right opposing quadrants (green regions in Fig. 7c), involving non-bilateral cell divisions at non-related positions within the lineage tree topology (Additional file 14: Figure S4). Third, and even more intriguing, we also observed “single quadrant bilateral symmetry,” where two symmetric clones originate from the same quadrant (brown regions in Fig. 7c). These findings contradict the initial assumptions [18] that simple bilaterally symmetric divisions should establish the bilaterally symmetric portions of the larval body, as observed for the 2d-112 and 4d somatic descendants in the larval hyposphere/trunk.

The disconnection between quadrant lineage and bilateral founders in medial regions suggests that the specification of these founders could be regulative (rather than mosaic)—triggered for example by a signaling source positioned in the plane of the bilateral symmetry. An obvious candidate for the signaling center is the 2d cell and its descendants, positioned in the anterior part of the dorsal hyposphere on the axis of the bilateral symmetry. These cells are well known for their organizing potential of *Platynereis* trunk [49], and significantly, the deletion of the 2d cell in *Capitella* leads to loss of bilateral symmetry in the head [53]. Interestingly, the regulative potential of the D quadrant does not seem to be limited to the C|D-A|B bilateral symmetry, but might also contribute to establishing the A|C bilateral symmetry, as demonstrated by its involvement in specification of the A and C quadrant-derived eyes in *Ilyanassa* [54].

### Conserved *six3+*, *otx+*, and *nk2.1+* head regions show distinct lineage behavior

Across Bilateria, the homeobox gene *six3* plays an evolutionary conserved role in the specification of the most apical body region, peripherally abutting the *otx*+ expression territory. The expression of *nk2.1* overlaps *six3* and *otx* expression on the ventral body side [14, 35, 55]. Mapping the expression of these genes on the tracked lineages, we observe an almost perfect match between expression regions and groups of cells with distinct (but internally consistent) lineage behavior. In particular, we notice that the combined expression of *six3*, *otx*, and *nk2.1* encompasses all bilateral founders that arise from the 1m-1121 and 1m-1122 micromeres and thus all lineages of subsequently differentiating cells with bilateral symmetry—at least transitorily. Among these, *six3* expression labels the more medially located 1m-1121 founders that are of different lineage in opposing quadrant, whereas *otx* labels the more lateral bilateral founders that stem from 1m-1122 micromeres, with similar lineages between quadrants. This observation opens up the possibility that *six3* and *otx* play an early role in determining the divergent lineage behavior of medial versus lateral bilateral founder cells during the spiral-to-bilateral transition. In line with this assumption, the medial micromeres that do not transition to bilateral symmetry are devoid of *six3*, *otx*, and *nk2.1* expression.

### The conserved *six3+* and *otx+* domains give rise to cholinergic brain neurons and head sensory organs

At later larval stages, *six3* and *otx* retain their antagonistic expression, yet transition clonal boundaries, so that the ring of *six3* expression expands to cover large part of the differentiating brain, whereas *otx* expression becomes restricted to few patches of cell in the periphery. *Six3* expression thus labels the bilateral sets of differentiating cholinergic neurons involved in the control of larval ciliary beating [12]. Interestingly, *six3* is expressed in cholinergic forebrain neurons in vertebrates [56] and in the central complex in the insect brain [57], which also contains cholinergic neurons [58]. A possible conservation of these cholinergic neuron types and their possible ancestral function can be tested by a broader comparative analysis of these neurons in other animals.

Mapping the gene expression atlas onto the larval *Platynereis* lineage yields another important insight. At 12 hpf, a small population of *phc2+* neuropeptidergic cells is found near the plane of bilateral symmetry in the dorsal embryo. Our atlas reveals that these cells are early representatives of a larger *phc2+* population that is constantly present around the apical organ at 34 hpf (Additional file 12: Figure S3). This population in turn expands to the population of *phc2+* cells present in the 48 hpf brain (termed “apical nervous system”; [32, 55]). Our integrated analysis reveals that, while some of these cells initially express *six3*, the gene is later turned off in these cells, so that the *phc2* expression domain largely matches the medial “hole” free of *six3* expression in the middle of the episphere [14], with the exception of few marginal cells co-expressing *phc2* and *six3* [32]. We and others have compared the *phc2-*expressing cells in the medial forebrain of invertebrates to the hypothalamus in vertebrates—which is likewise surrounded by the *six3*-expressing cholinergic forebrain [12].

In contrast to the *six3+* bilateral founders, the *otx+* bilateral founders proliferate heavily during later stages and differentiate much later, into adult eyes and optic lobes [36], indicating that the *otx+* cells contribute to head sensory organs rather than cerebral ganglia. Together, these findings indicate that the ring of *six3* expression in the larval episphere gives rise to large part of the cerebral ganglia, whereas sensory organs and associated brain centers emerge from more lateral *otx*+ territory and medial neurosecretory centers from the most apical region devoid of *six3* expression.

### Lineage comparison to other spiralians

Finally, our data allows comparing the lineage of similar cells with particular identities between *Platynereis* and other spiralian species. For example, the lineage of the accessory trochoblasts is traditionally reported as 1m-12 [23, 26]. The descendants of 1m-12 form differentiated accessory prototroch cells (1m-122 and 1m-1212), characterized by a tight association with primary prototroch cells. Interestingly, their cell lineage in *Platynereis* is different from accessory trochoblasts in the polychaetes *Amphitrite* and *Podarke* (1a-2222, 1c-1222) but partially similar to the mollusk *Dentalium* [10]. In *Platynereis*, the progeny of 1d-12 does not only give rise to one accessory prototroch cell, but some migrate posteriorly to contribute to the antero-median part of the dorsal hyposphere, as reported for other polychaetes [10] and references therein. The *Platynereis* cell lineage (1c/d-11221) migrating laterally and forming the “head kidneys” posteriorly to the prototroch agrees with previous reports for *Nereis* [18].

The spiralian apical organ is assumed to generally derive from the apical rosette cells, although actual cell lineage studies are mostly lacking [59]. The cells forming the apical tuft in *Platynereis* are 1c-1111/1c-1112 (the ampullary cells) and 1d-1111/1d-1112 (the large apical dorsal cell and first apical axon-projecting cell), and thus indeed derived from apical rosette. Similarly, in the mollusk *Dentalium*, the 1a^111^–1d^111^ and 1a^1121^–1b^1121^ contribute to the apical organ, with the apical tuft developing from 1c^1111^ and 1d^1111^ [10].

### Outlook

Our data yields first insight into the interplay between cellular lineage and gene regulatory networks in spiralian development, spanning the transition from embryonic and larval rotational symmetry to the bilateral symmetry of the juvenile. Future lineage data extending beyond 32 hpf will be integrated with refined expression atlases generated through Profiling by Signal Probability Mapping (ProSPr [25];), and with single-cell expression data mapped onto the expression atlases for reference embryonic and larval stages [32]. This will allow the identification of candidate signals and receptors, as well as the gene regulatory networks establishing bilateral symmetrical behavior and cell fates in spiralian development.

## Materials and methods

### Animals

The larvae of *Platynereis dumerilii* were obtained from the breeding culture at EMBL Heidelberg.

### Injections and time-lapse imaging

The injections of mRNAs encoding for H2A-RFP (courtesy of the Gilmour lab, EMBL Heidelberg) and Lyn-EGFP proteins [27] were performed as described previously [60]. For tracking axonal projections, *lifeact-egfp* mRNA [61] was injected at concentration 200 ng/μl into a given blastomere of embryos injected previously at 1-cell stage with mRNA encoding H2A-RFP protein.

The injected embryos were kept in filtered seawater at 18 °C until the desired developmental stage was reached. Selected embryos were then transferred in ~ 2 μl of sea water into 40 °C 0.8% low-melting agarose (A9414, Sigma-Aldrich), briefly mixed by pipetting up and down and quickly transferred in ~ 20 μl agarose to the microscopy slide with 150 μm spacer on each side (3 layers of adhesive tape Magic™Tape, Scotch®). Before the agarose fully solidified (within ~ 15 s), the embryos were covered by a coverslip and oriented to the apical position for imaging. Seawater was added from the side of the slide to entirely fill the slide chamber. To avoid drying out, the coverslip was sealed using mineral oil. Embryo 1 and embryo 2 were imaged using a Zeiss Axio Imager, × 40 oil immersion objective, with 0.48-μm XY resolution and 1-μm z resolution. Other embryos were imaged with a M1 fluorescent microscope or Leica TCS SPE confocal microscope with × 40 oil immersion objective. The imaging on the confocal microscope was performed with 1.5-μm z resolution for all embryos, and 0.414-μm xy resolution (embryo 3), 0.384-μm xy resolution (embryo 10), and 0.387 μm (embryo 11). The formal time resolution of the recordings is as follows: 6 min (embryo 1), 12 min (embryo 2), 12 min (embryo 3), 9 min (embryo 10), and 8 min (embryo 11). However, due to the heat produced by the imaging and instability of the temperature in the microscopy room, the imaging time does not directly relate to the developmental time. To compensate for this, the live-imaging movies were calibrated using the episphere nuclei counts from embryos freely developing in 18 °C sea water and fixed at given developmental time (5, 10, 12, 14, 16, 20, 24, and 30 hpf), stained with DAPI and imaged using confocal microscopy. The calibrated developmental time is time-stamped on the reference lineage movies 1 and 2 (Additional files 3 and 6, as well as the Z-projections of 4D recording of each embryo, available in the online data repository [28]). After imaging, the embryos were quickly assessed for viability (coordinated ciliary beating, spiral swimming, gross morphology) using wide-field microscopy and immediately fixed. Misdeveloping embryos were excluded from subsequent analyses.

### Tracking and comparing the cell lineage across multiple embryos

The live-imaging movies were manually tracked using a custom-made tracking macro in ImageJ/FiJI [29]. We used the nuclei count of the episphere in embryos precisely fixed at several time points to calibrate the developmental time in the movies. Due to a high density of nuclei at later stages, we were able to reliably track until about 32 hpf. At early developmental stages, we use the standard spiralian nomenclature of the cells according to [62]. After 6 hpf, even for non-spiral cell divisions, we use the index 1 for the more anterior and index 2 for the more posterior daughter cell until around 10 hpf. After 10 hpf, we use indices “a” and “b” instead of “1” and “2,” to emphasize that the cells do not divide in the spiralian cleavage pattern any more. Within the spiral cleavage phase, we use the abbreviated form 1m-xyz to collectively refer to all four quadrant homologs (i.e., cells 1a-xyz, 1b-xyz, 1c-xyz, and 1d-xyz).

#### Comparing the lineage of multiple embryos

To compare the cell lineage across different embryos, a simple algorithm automatically identifying corresponding cells in each tracking dataset and highlighting the differences was used (Additional file 9: Figure S1F): First, the corresponding cells were manually assigned in the first frames of the 4D recordings, representing the roots of the lineage trees. Subsequently, at the earliest following cell division, several “features” (relative spatial position of the daughter cells, subsequent cell cycle length, and number of descendants of each of the two daughter cells) were extracted. These features are then used to generate a feature matrix for each pair of daughter cell in various embryos. The feature matrices are then compared among daughter cells from different embryos using weighting coefficients (determined arbitrarily) that results in a similarity score (Additional file 9: Figure S1F). The cells with the highest similarity score are then assigned to be corresponding cells between the two embryos and thus provide the new rooting point for the next repetition of the same procedure. The decision procedure is then performed for the following cell division, identifying the corresponding cells, and proceeds throughout the entire lineage trees until all corresponding cells are identified.

As the 4D recordings cover the first ~ 34 hpf of development, there are still many cell divisions to come after the last frame of the recordings. Due to the increasing asynchrony between division timing (Additional file 9: Figure S1H), corresponding cell divisions can occur before the end of the 4D recording in one embryo but after the last frame of the 4D recording in another embryo, leading to a false difference in the comparison (Additional file 9: Figure S1G-G’). To avoid this problem of overestimating the number of differences, the recordings were compared at 30 hpf, and the remaining recording frames were used “known divisions to come.” We estimated a safe time point to compare the recordings to be 30 hpf (~ 3 h before the last recorded time frames), since the average of maximum difference in cell division across the three time-lapse recordings increases with developmental time and reaches about 2.5 h between 30 and 34 hpf (Additional file 9: Figure S1H).

### Whole-mount mRNA in situ hybridization

The mRNA in situ hybridization was performed as described in [63] with the following modifications: For developmental stages earlier than 12 hpf, the embryos were washed twice 4 min with calcium/magnesium-free sea water [64] prior to fixation. For developmental stages younger than 24 hpf, the embryos were acetylated: After the digestion in proteinase K and two washes with freshly prepared 2 mg/ml glycine in PTW (1× phospate-buffered saline with 0.1% Tween-20), the embryos were incubated 5 min in 1% triethanolamine in PTW, then 3 min in 1% triethanolamine with 0.2% acetic anhydride followed with 3 min of 0.4% acetic anhydride in 1% triethanolamine. The prehybridization, hybridization, and SSC washes were performed at 63 °C. The hybridization mixture: 50% Formamide (Sigma-Aldrich, F9037), 5× SSC pH 4.5, 50 μg/ml Heparin (Sigma-Aldrich, H3149), 0.025% Tween-20 (Sigma-Aldrich, P9416), 50 μg/ml Salmon Sperm DNA (Sigma-Aldrich, D9156), 1% SDS. The antisense mRNA probes for *chat* and *elav* [65]; *syt*, *tph*, *phc2*, and *nk2.1* [33]; *vacht* [13]; *otx* [66]; *six3/6* [35]; and *vglut* [37] were DIG-labeled using DIG RNA Labeling Mix (Roche, 11 277 073 910). Typically, 10–20 embryos were processed per developmental stage and gene, and 2–3 embryos imaged using confocal microscopy. For mapping gene expression onto the reference movies, 2–3 embryos were imaged after WMISH using the reflection of NBT/BCIP precipitate [67] and counterstained with DAPI to reveal nuclei and acetylated tubulin to facilitate orientation based on the ciliary band. The mouse anti-acetylated Tubulin antibody (Sigma, T6793) was used at 1:500 dilution and detected by secondary Alexa488-conjugated anti-mouse antibody (Jackson ImmunoResearch, 115-546-062, 1:500). Then, the DAPI channel was used and carefully compared to the 3D stack of the reference movie at corresponding developmental stage. Corresponding nuclei were identified based on their shape, staining intensity, and relative position.

## Supplementary information

**Additional file 1.** The ImageJ/FIJI macro package for visualization of the lineages using the tracking data and the lineage movies. The macro package allows visualizing the track on top of the lineage movies, finding a given cell by the lineage name or the reference ID name, changing colors of a given cell, its descendants and/or ancestors. Documentation is provided in Additional file 2.

**Additional file 2. **The documentation to the Additional file 1: *PduLineageMacroPackage.ijm* macro package contains instructions how to install the macro package and use it functions.

**Additional file 3.** The movie is a z-projection of combined live-imaging recordings of Embryo 1, Embryo 2 and Embryo 3) and shows the development of the episphere from ~ 6 hpf until ~ 33 hpf. Could be opened by the ImageJ/FIJI software [29]. The original 4D recordings of the embryos are available in online data repository [28].

**Additional file 4.** The track of the Additional file 3: Reference_Lineage_Movie1.tif contains the xyzt coordinates of the cells, their lineage names and reference ID names. The coloring scheme of the track corresponds to the coloring of bilateral founders in Fig. 5A-A’. The track can be visualized on top of the movie using the Additional file 2.

**Additional file 5.** This file is a 7z archive of the lineage trees of the Reference_Lineage_Movie1. The particular tree files are in scalable vector graphics format (.svg). The coloring scheme of the track corresponds to the coloring of bilateral founders in Fig. 5A-A’.

**Additional file 6.** The movie is a z-projection of combined live-imaging recordings of Embryo 1 and Embryo 10) and shows the development of the episphere from ~ 6 hpf until ~ 33 hpf. Could be opened by the ImageJ/FIJI software [29]. The original 4D recordings of the embryos are available in online data repository [28].

**Additional file 7.** The track of the Additional file 6: Reference_Lineage_Movie2.tif contains the xyzt coordinates of the cells, their lineage names and reference ID names. The coloring scheme of the track corresponds to the coloring of bilateral founders in Fig. 5A-A’. The track can be visualized on top of the movie using the Additional file 2.

**Additional file 8.** This file is a .7z archive of the lineage trees of the Reference_Lineage_Movie1. The particular tree files are in scalable vector graphics format (.svg). The coloring scheme of the track corresponds to the coloring of bilateral founders in Fig. 5A-A’.

**Additional file 9: Figure S1.** Comparing the cell lineage among multiple embryos. This supplementary figure provides details about the comparison of the cell lineage among multiple embryos and identifying corresponding cells. (A-D’) The comparison between the clonal domains revealed by injections of *h2a-rfp* mRNA into a single blastomere and the clonal domain of the corresponding blastomere highlighted in red using the reference lineage movie at 32 hpf. (E) Comparison of the clonal domains originating from the cells present at 13 hpf in three different embryos. (F) Identification of corresponding cells between embryos: Multiple features (number of descendants, time till next cell division, relative cell position of each daughter cell) are extracted from the tracking information at each cell division. The feature arrays are compared between embryos to score the similarity and identify corresponding cells. For more details, see *Materials and methods*. (G-G’) The problem of assessing the differences between incomplete lineage trees: Due to the asynchrony in cell division timing, some of the corresponding divisions can happen after the last frame of a given movie (dashed line) – e.g. the magenta lineage within the blue domain in Lineage 1 divides later and therefore does not represent a real difference in cell division pattern. In contrast, the red cells within the green sublineage do not divide in Lineage 2 and therefore represent a real difference. (H) The average maximal difference in timing of corresponding cell divisions across three embryos increases with time, reaching around 2.5 h at 30 hpf. The maximal difference in timing was calculated as the difference between the time point at which the corresponding division occurred earliest among the three embryos, and the latest among the three embryos. The average difference was calculated from all corresponding cell divisions happening within the given hour post fertilization.

**Additional file 10: Figure S2.** The consensus lineage tree of the episphere development from fertilization until 30 hpf. The previously described early cell lineage is highlighted in blue [26]. The black branches represent a consensus of three embryos. If one embryo differs from the remaining two, the tree topology based of the two embryos is shown in gray. The horizontal error bars at cell division time points represent the minimal and maximal time point observed for that cell division. The numbers rNNN (eg. r214) above the branch represent the unique cell ID that can be used to find/label the cell within the ImageJ/FIJI macro (Additional file 1: PduLineageMacroPackage.ijm). The annotations of the known differentiated cell types from Table 1 are shown at the end points of the branches. See Fig. 2d for more details.

**Additional file 11: Table S1.** The table contains the list of all cells in the consensus lineage tree (Additional file 10), including the lineage name and the reference ID.

**Additional file 12: Figure S3.** A gene expression atlas of the episphere between 12 and 34 hpf. Contains whole-mount RNA in situ hybridization expression pattern for 23 genes at 7 stages (12, 14, 16, 20, 24, 30 and 34 hpf). (A) The expression of bHLH transcription factors. (B) The expression of *klf* and *prox* transcription factors. (C) The expression of neuronal differentiation markers. All panels are apical views with dorsal side on the top of the panel. Embryos were counterstained with DAPI to reveal the nuclei, axonal projections and ciliary band (green) were visualized using anti-acetylated-tubulin antibody staining.

**Additional file 13: Table S2.** The list of genes in the WMISH atlas between 12 and 34 hpf (Additional file 12).

**Additional file 14: Figure S4.** Establishment of bilateral clonal domains. This figure contains the details of the cell divisions and lineage of the bilateral founder cells. (A) The bilateral founders, descending from the 1 m-1122 cells, located more laterally, are generated in a perfect bilateral symmetry, reflected by a bilaterally symmetrical arrangement of the resulting lateral clones. All descendent lineages show full bilateral symmetry, as is apparent from the equivalent lineage history of right and left counterpart clones (bottom panel). (B-C) For the bilateral founders in the dorso-medial (B) and ventro-medial (C) regions descending from 1 m-1121 sublineages, the lineage history of the left and right founder is very different. These founders originate at different branches of the quadrant homologue lineage tree and in some cases even differ in the lineage depth (light green, red, and dark green clones in panel B; light green clones in panel C). Two bilateral founder pairs - 1a-1121211 and 1a-1121121 (light and dark blue clone in panel C) and 1b-12111aa and 1b-121121b (dark green in D) originate from single quadrants. Note, that the cell divisions occurring at the lateral-most edge of this largely asymmetrical medial domain produce again symmetrical clones (sand and light brown clones in panel B). (D) The origin of A|C symmetry: The cells 1 m-12 divide spirally to produce accessory prototroch cells 1 m-122 and 1 m-1212. Subsequent cell divisions within 1c-12 and 1a-12 clone occur in a bilateral mode resulting in fully bilateral domains stemming from the A and C quadrant.

**Additional file 15: Figure S5.** The dynamics of early *otx* expression. This figure shows the developmental expression of *otx* between 6 and 12 hpf mapped onto the cell lineage and reveals the dynamic switching of *otx* during episphere development. *Otx* expression visualized by WMISH (column next to the lineage trees) was mapped on lineage movie and lineage tree at indicated stages. Corresponding nuclei between the stained embryos and the movie frame (horizontal arrows) were identified manually at these stages. The vertical arrows indicate the theoretical clonal expansion of *otx*-positive cells at later stages. The comparison of such theoretical clonal expansion of *otx*-expressing cells at different stages to real expression pattern at a given stage (WMISH panel next to the lineage trees) shows that *otx* expression is not clonal and that the gene is dynamically switched on/off between cell cycles. The dynamic on/off switching of early *otx* expression is apparent in the lineage trees on the right-hand side, where the *otx*-expressing cells (in red) do not form a continuous lineage.

## Data Availability

All data presented in this study are included in the published article and the additional files and public data repository: 10.6084/m9.figshare.c.4659302 [28].
