# Supplementary material for: From spiral cleavage to bilateral symmetry: the developmental cell lineage of the annelid brain
Source: BMC Biol. 2019 Oct 22;17:81. doi: 10.1186/s12915-019-0705-x (PMC6805352; doi:10.1186/s12915-019-0705-x)
Supplement: Supplementary file 2 — Additional file 2. The documentation to the Additional file 1: PduLineageMacroPackage.ijm macro package contains instructions how to install the macro package and use it functions. [file 12915_2019_705_MOESM2_ESM.pdf]

Guide for exploring *Platynereis* episphere cell lineage

This guide provides information how to use the lineage macro package for exploring the episphere cell lineage of *Platynereis dumerilii.* It is a part of a published article: Vopalensky P, Tosches MA, Achim K, Handberg-Thorsager M, Arendt D: **From spiral cleavage to bilateral symmetry: the developmental cell lineage of the annelid brain.** *BMC Biol, 2019*

1) Necessary files

To explore the *Platynereis* lineage, you will need ImageJ/FIJI and three files:

- A movie of *Platynereis* episphere development, provided as:
  - **Additional file 3**: *Reference_Lineage_Movie1.tif*
  - **Additional file 6**: *Reference_Lineage_Movie2.tif*
- Lineage macro package provided as:
  - **Additional file 1:** *PduLineageMacroPackage.ijm*.
- The lineage track of *Platynereis* episphere development, provided as:
  - **Additional file 4:** *Reference_Lineage_Movie1_Track.txt*
  - **Additional file 7:** *Reference_Lineage_Movie2_Track.txt*

More lineage 4D recordings of individual embryos and their tracks are available in the online repository: Vopalensky P, Tosches MA, Achim K, Handberg-Thorsager M, Arendt D: **From spiral cleavage to bilateral symmetry: the developmental cell lineage of the annelid brain. Supplementary datasets**; figshare 2019: [https://doi.org/10.6084/m9.figshare.c.4659302]

2) Installing the Lineage macro package to FIJI

Open FIJI, and install the macro package by going to Plugins/Macros/Install..., then select the Lineage macro package file (*PduLineageMacroPackage.ijm).* If everything works well, you should get 5 more tools in your FIJI screen: LinView, Cell name, Find a cell, Draw tree and Save Coloring.

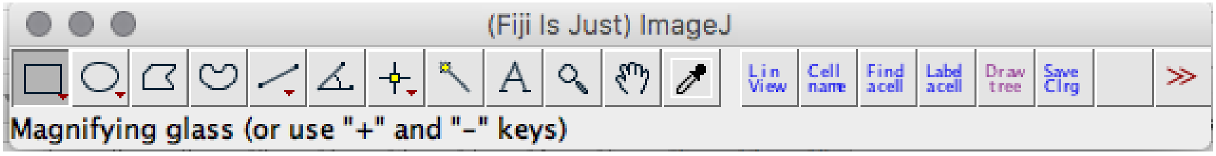

3) Opening a movie

Now open the z-projection movie (e.g. *Reference_Lineage_Movie1.tif*) in FIJI by going to File/Open and find the file. The movie should open in a new FIJI window. You can navigate through the movie by the slider under the images.

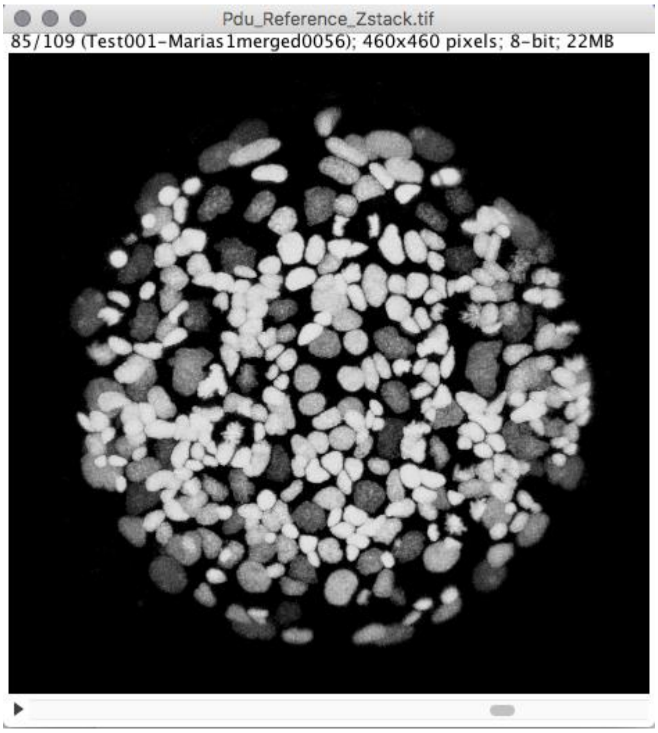

4) Loading the lineage track onto the movie

To load the lineage track on top of the movie, press LinView icon in FIJI panel and the following window will pop up:

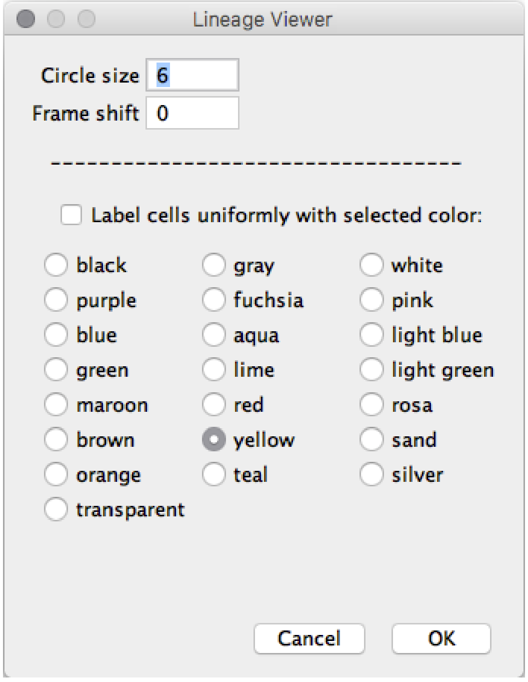

The circle size defines how large the circles defining your cells will appear; values between 6 and 12 are good. Leave Frame shift set to 0.
The lineage track file contains the information about cell coloring. If you want to label all cells uniformly, tick the "Label cell uniformly…" line and select a color to be used. If you do not tick this option, the colors will be loaded from the track file.

After clicking OK, select the .txt file containing the corresponding lineage track: e.g. *Reference_Lineage_Movie1_Track.txt*

Now, the lineage track should be loaded on top of your movie and you should see the nuclei labeled with colored circles:

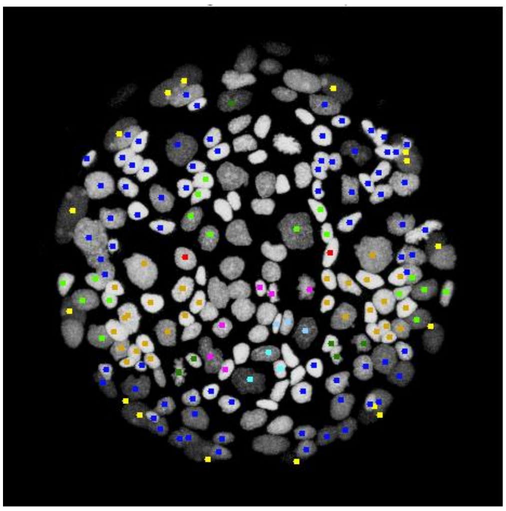

5) Selecting a cell

To select a "cell" (circle), either long left-click on a circle or hold the left ALT key and left click on the circle. After being selected, the circle will get a dashed outline:

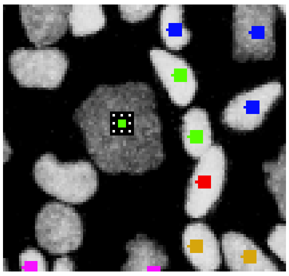

With a cell selected, you can **get the Cell name** by clicking on "Cell name" in the FIJI menu.

A small log-window should appear in which you can read the cell name:

#######
r923-61
1d-11211112ab
x: 270 y: 204 z: 12 t: 65

The “r923-61” is a **reference number** (rID) that is unique to every cell in a given mitotic cell cycle. The suffix “-61” indicates the frame of the movie in which the cell originates.
Under the reference name, you see the **lineage name** (1d-11211112ab).
The **coordinates** in the last line just specify the x,y,z and t coordinates of the cell you selected.

6) Labeling cells, ancestors and descendants with different colors

With a cell being selected, you can also label the cell and/or its ancestors and/or its descendants by clicking on Label a cell in the FIJI menu. The following menu will appear:

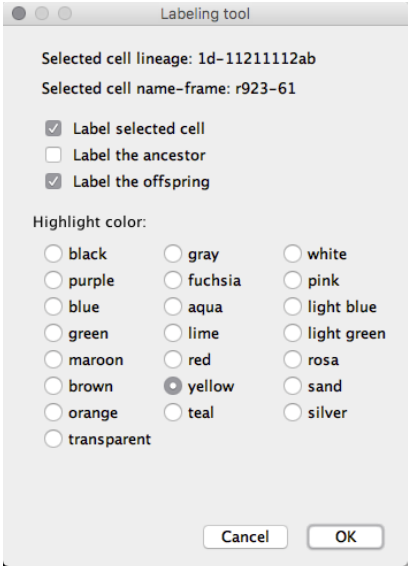

You can tick whatever option (just the cell, ancestor, offspring or all of these) and then select the color you want for labeling. In the following example, the offspring of r923-61 is relabeled from green to white.

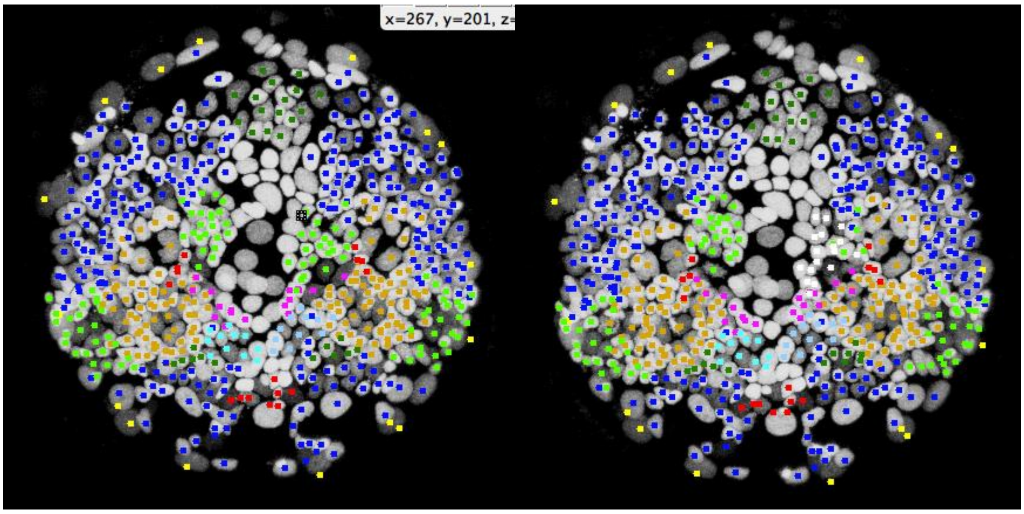

7) Finding a cell by its name.

Sometimes you do not want to select a cell by clicking on it, but rather do the opposite – find the cell based on its name. To do so, find the name of the cell e.g. by looking at the

consensus lineage trees that contains the reference name for all cells in the lineage. In the following picture, you see a very small part of the lineage tree with cells labeled with a reference name (the top-most being r1114). Some of the cells in the lineage tree have a special line with a number that corresponds to the number in Table 1 and a short annotation. For example, cell with a reference ID r1113 is the *chat* positive, left lateral bigger cell #51 in Table 1.

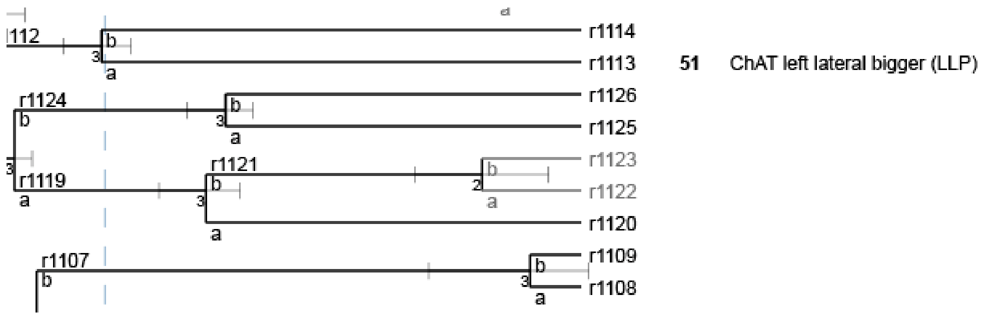

To find the cell r1113, click on "Find a cell" in the FIJI panel and the following window will appear:

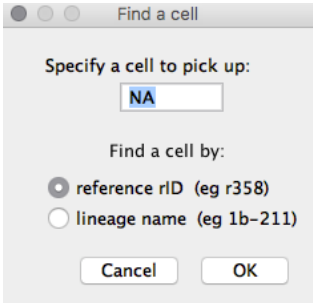

Enter r1113 in the textbox "Specify a cell to pick up" and select "reference rID". After clicking OK, you should get the frame of the movie with the cell r1113 selected by dashed line:

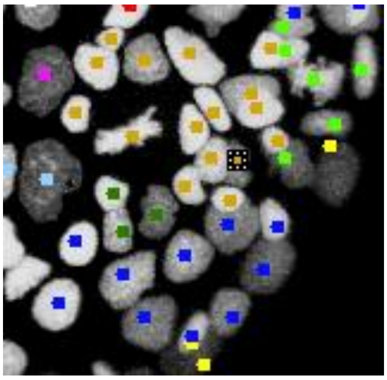

Now, since the cell got selected by the tool, you can also get the lineage name by clicking "Cell name" and recolor as you wish (see chapter 5 and 6):

r1113-69
1d-112112222aa
x: 358 y: 294 z: 24 t: 69

7) Labeling a cell based on its name

In case you do not want to label a cell by selecting it, but rather by directly entering its lineage name. To do so, make sure no cell is selected – by clicking anywhere outside any circle. Then click on "Label a cell" in the FIJI panel and in the dialogue window enter the lineage name:
eg: 1d-112112222aa.

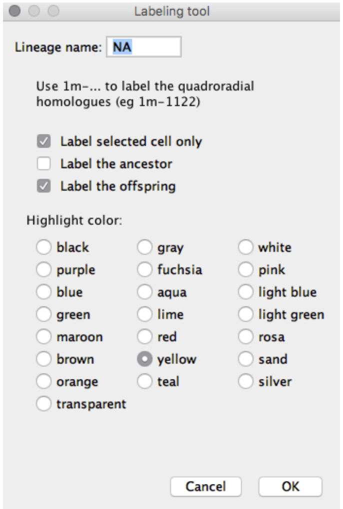

This window also allows you to label the corresponding cells from all four quadrants in the same color. Imagine you want to label all 1a-1122, 1b-1122, 1c-1122 and 1d-1122 in the same color, then just enter 1m-1122 in the window. This function is useful for analyzing the relationship between spiral and bilateral symmetry.

8) Saving a re-colored track

If you colored your track according to your wishes, you can also save the new track by clicking on "Save clrg" in the FIJI panel and specifying the saving location and the file name. The recolored track gets saved as a new Track file with changed colors.

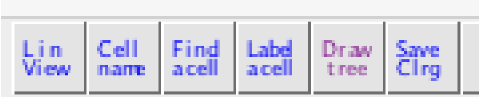

This operation is not optimized and therefore could take a few minutes, depending on the computational power of your computer.

9) Drawing trees from a track

If you want to draw a tree from your track, including the coloring, click on "Draw tree" in the FIJI panel and select a Track file. The tool identifies how many independent trees are in the track and saves them as independent .svg files (to be able to edit in Adobe Illustrator). The trees are saved in the same folder as the Track file used for generating the trees. For example, there will be 8 independent cell lineage trees generated from the *Reference_Lineage_Movie1_Track.txt*, because the first frame of the movie contains 8 different cells and the tracks starts with 8 individual lineages.
