# Supplementary figures and images for: From spiral cleavage to bilateral symmetry: the developmental cell lineage of the annelid brain
Source: BMC Biol. 2019 Oct 22;17:81. doi: 10.1186/s12915-019-0705-x (PMC6805352; doi:10.1186/s12915-019-0705-x)

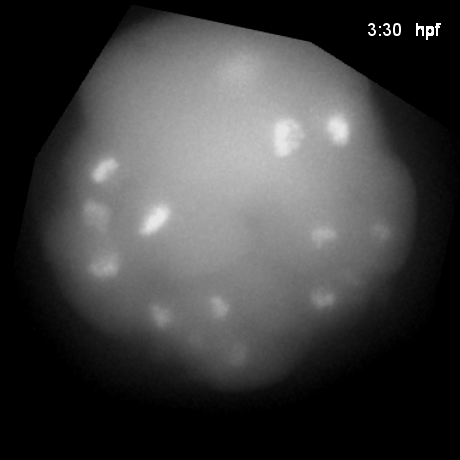

Supplement: Supplementary file 3 — Additional file 3. The movie is a z-projection of combined live-imaging recordings of Embryo 1, Embryo 2 and Embryo 3) and shows the development of the episphere from ~ 6 hpf until ~ 33 hpf. Could be opened by the ImageJ/FIJI software [29]. The original 4D recordings of the embryos are available in online data repository [28]. [file 12915_2019_705_MOESM3_ESM.tif]

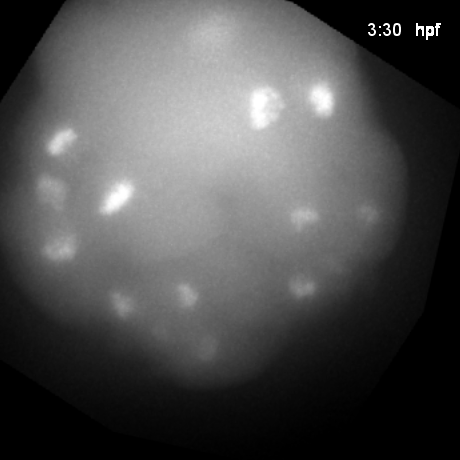

Supplement: Supplementary file 6 — Additional file 6. The movie is a z-projection of combined live-imaging recordings of Embryo 1 and Embryo 10) and shows the development of the episphere from ~ 6 hpf until ~ 33 hpf. Could be opened by the ImageJ/FIJI software [29]. The original 4D recordings of the embryos are available in online data repository [28]. [file 12915_2019_705_MOESM6_ESM.tif]

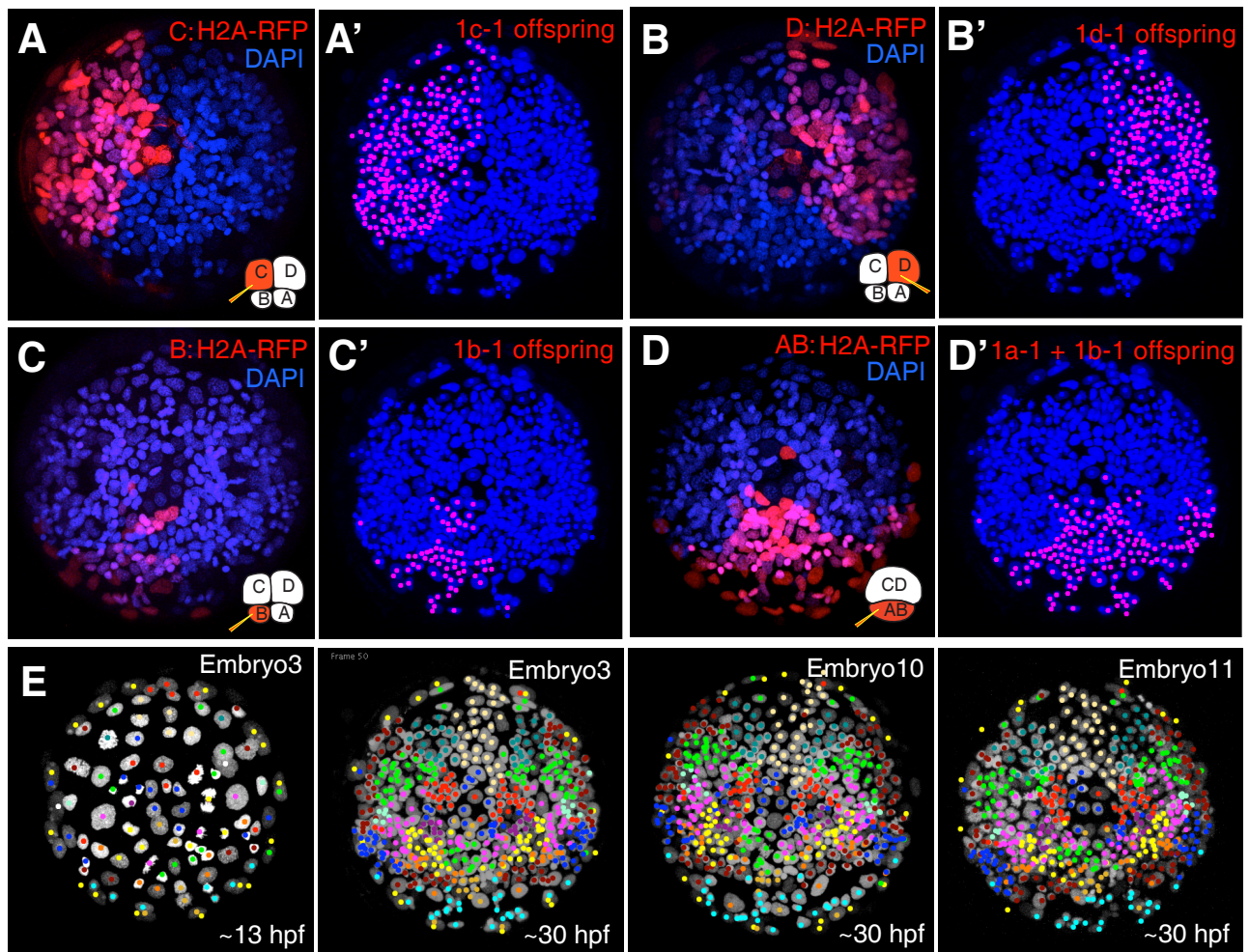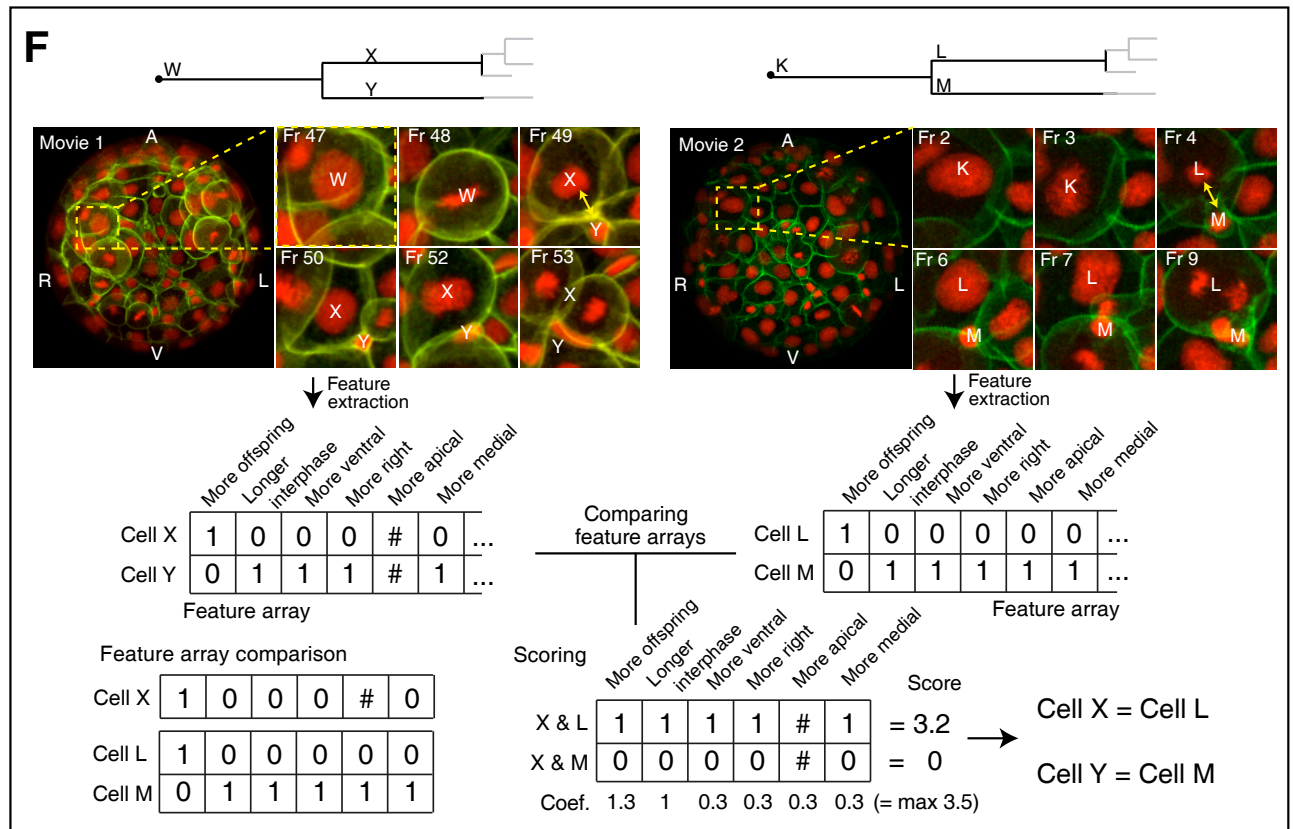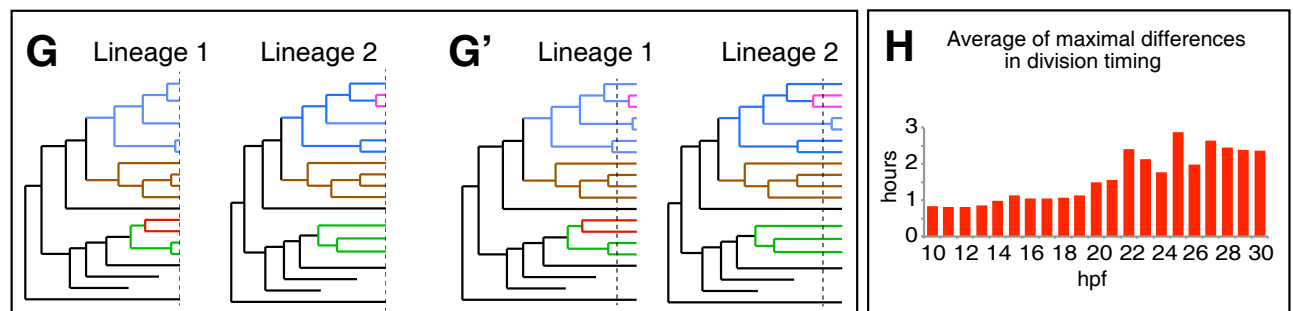

Supplement: Supplementary file 9 — Additional file 9: Figure S1. Comparing the cell lineage among multiple embryos. This supplementary figure provides details about the comparison of the cell lineage among multiple embryos and identifying corresponding cells. (A-D’) The comparison between the clonal domains revealed by injections of h2a-rfp mRNA into a single blastomere and the clonal domain of the corresponding blastomere highlighted in red using the reference lineage movie at 32 hpf. (E) Comparison of the clonal domains originating from the cells present at 13 hpf in three different embryos. (F) Identification of corresponding cells between embryos: Multiple features (number of descendants, time till next cell division, relative cell position of each daughter cell) are extracted from the tracking information at each cell division. The feature arrays are compared between embryos to score the similarity and identify corresponding cells. For more details, see Materials and methods. (G-G’) The problem of assessing the differences between incomplete lineage trees: Due to the asynchrony in cell division timing, some of the corresponding divisions can happen after the last frame of a given movie (dashed line) – e.g. the magenta lineage within the blue domain in Lineage 1 divides later and therefore does not represent a real difference in cell division pattern. In contrast, the red cells within the green sublineage do not divide in Lineage 2 and therefore represent a real difference. (H) The average maximal difference in timing of corresponding cell divisions across three embryos increases with time, reaching around 2.5 h at 30 hpf. The maximal difference in timing was calculated as the difference between the time point at which the corresponding division occurred earliest among the three embryos, and the latest among the three embryos. The average difference was calculated from all corresponding cell divisions happening within the given hour post fertilization. [file 12915_2019_705_MOESM9_ESM.pdf]

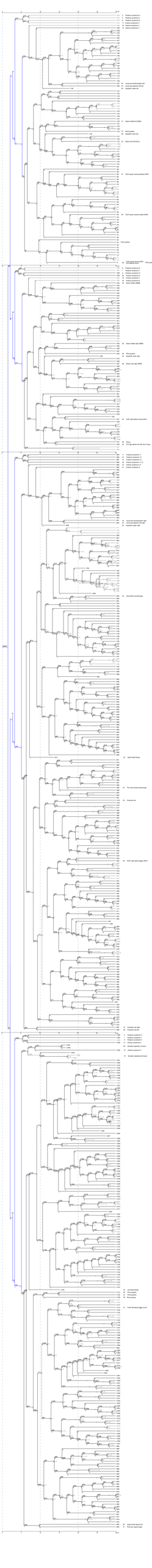

Supplement: Supplementary file 10 — Additional file 10: Figure S2. The consensus lineage tree of the episphere development from fertilization until 30 hpf. The previously described early cell lineage is highlighted in blue [26]. The black branches represent a consensus of three embryos. If one embryo differs from the remaining two, the tree topology based of the two embryos is shown in gray. The horizontal error bars at cell division time points represent the minimal and maximal time point observed for that cell division. The numbers rNNN (eg. r214) above the branch represent the unique cell ID that can be used to find/label the cell within the ImageJ/FIJI macro (Additional file 1: PduLineageMacroPackage.ijm). The annotations of the known differentiated cell types from Table 1 are shown at the end points of the branches. See Fig. 2d for more details. [file 12915_2019_705_MOESM10_ESM.pdf]

**A**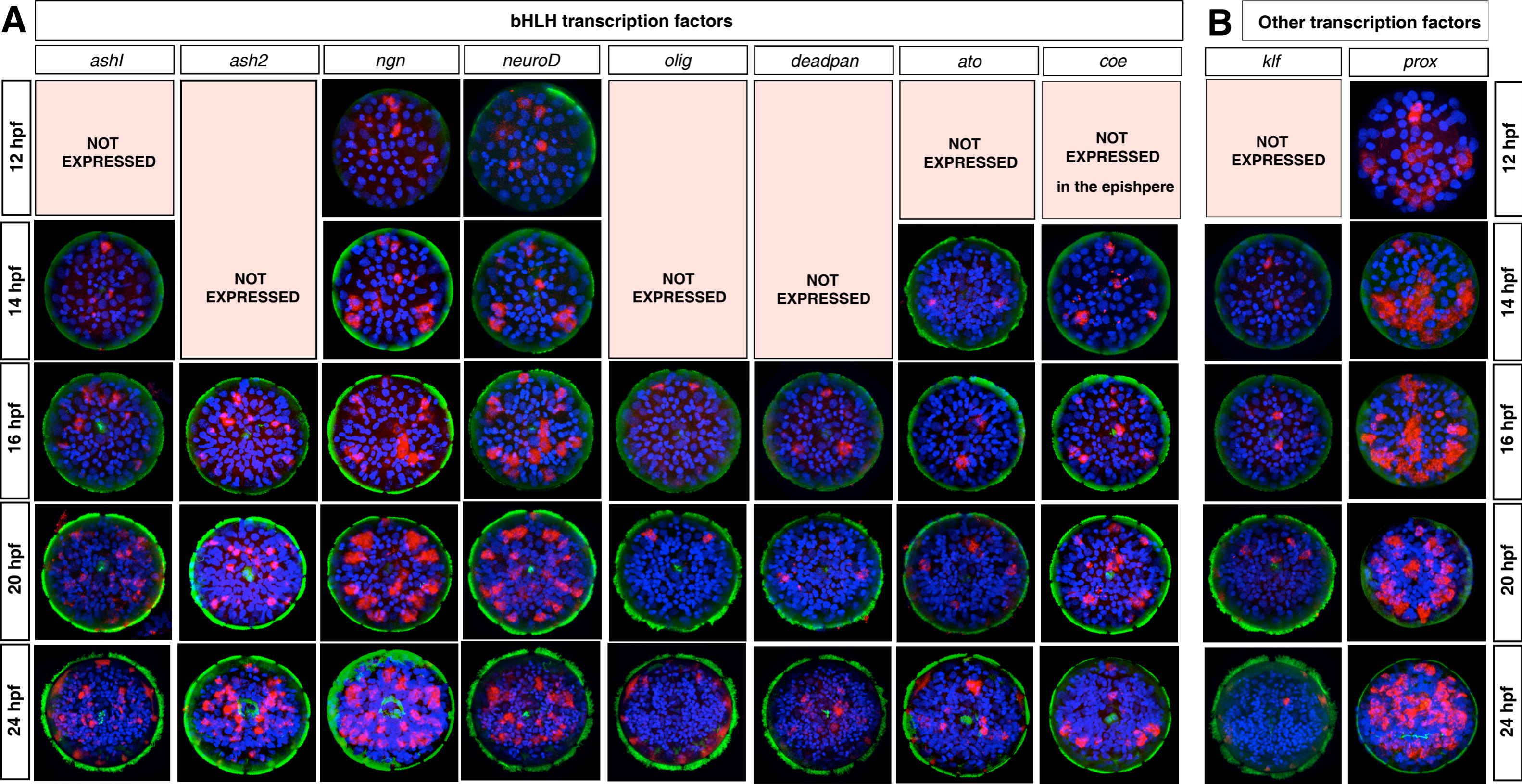**C**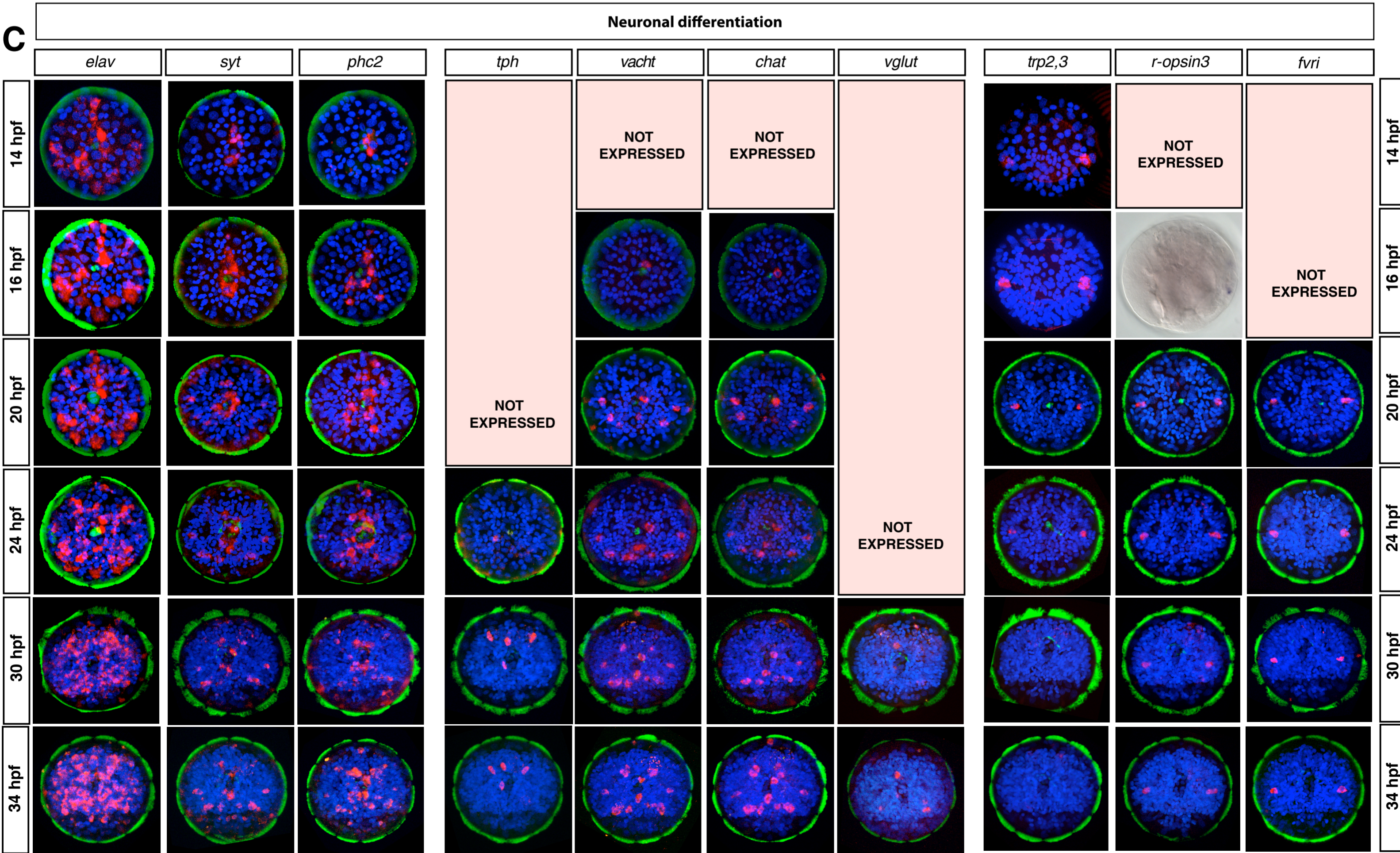

Supplement: Supplementary file 12 — Additional file 12: Figure S3. A gene expression atlas of the episphere between 12 and 34 hpf. Contains whole-mount RNA in situ hybridization expression pattern for 23 genes at 7 stages (12, 14, 16, 20, 24, 30 and 34 hpf). (A) The expression of bHLH transcription factors. (B) The expression of klf and prox transcription factors. (C) The expression of neuronal differentiation markers. All panels are apical views with dorsal side on the top of the panel. Embryos were counterstained with DAPI to reveal the nuclei, axonal projections and ciliary band (green) were visualized using anti-acetylated-tubulin antibody staining. [file 12915_2019_705_MOESM12_ESM.pdf]

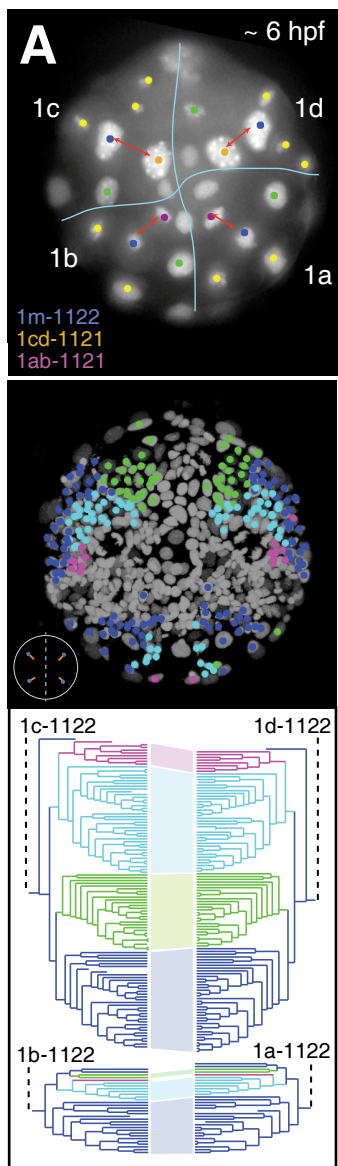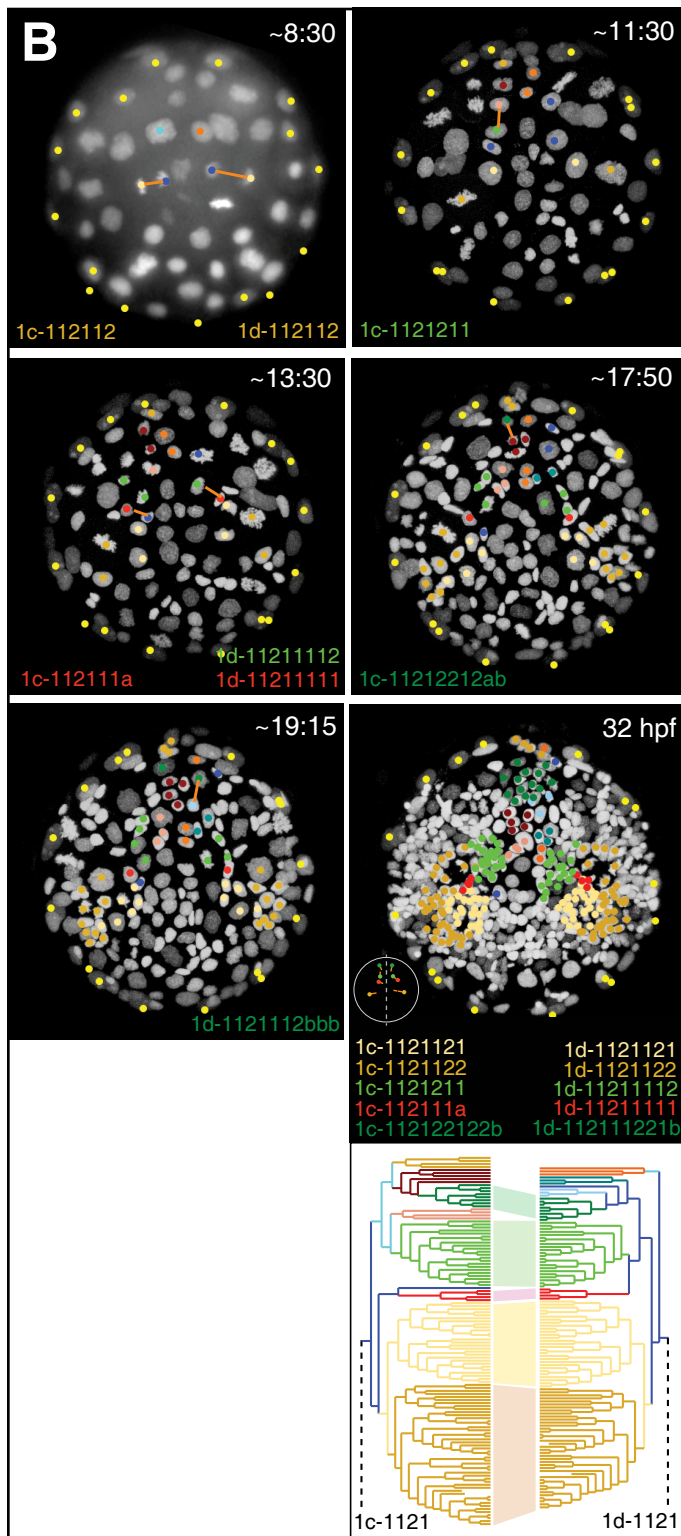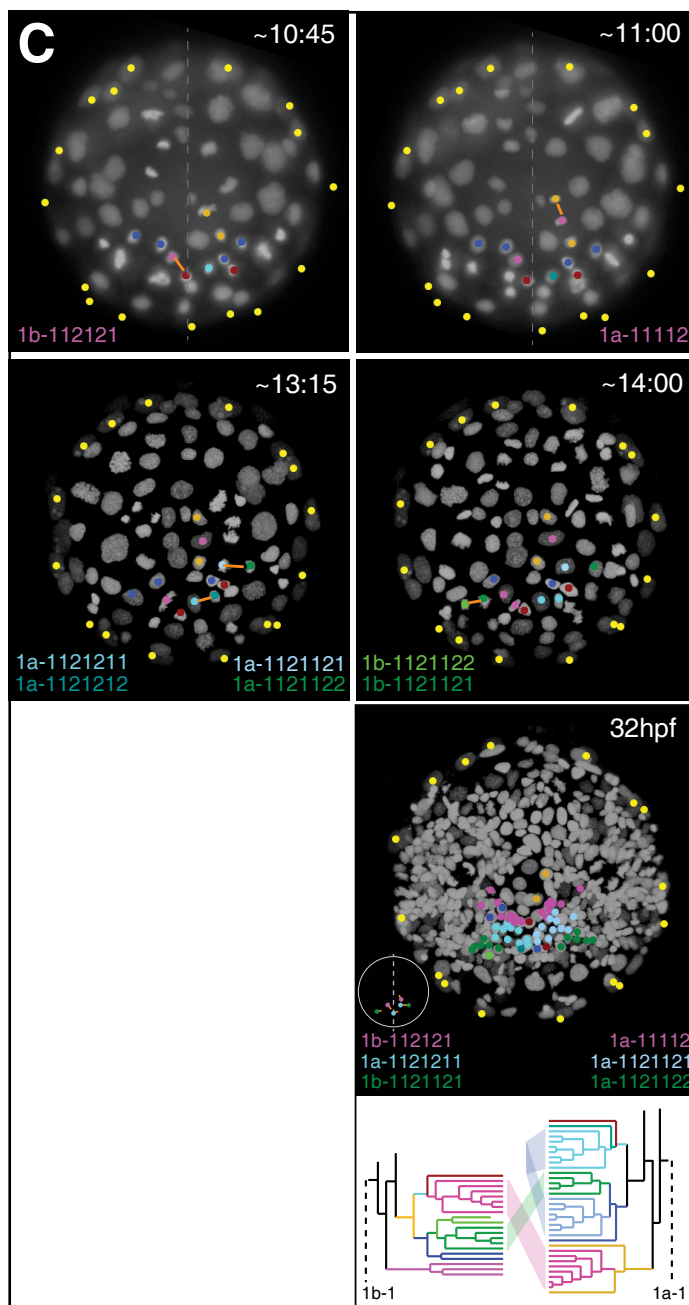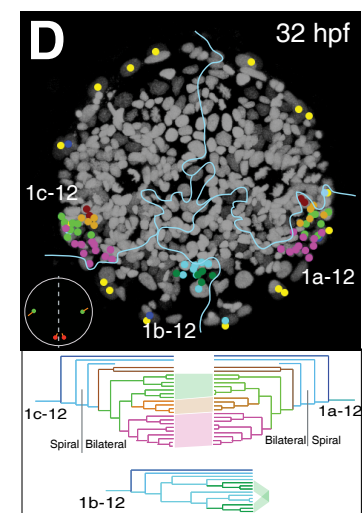

Supplement: Supplementary file 14 — Additional file 14: Figure S4. Establishment of bilateral clonal domains. This figure contains the details of the cell divisions and lineage of the bilateral founder cells. (A) The bilateral founders, descending from the 1 m-1122 cells, located more laterally, are generated in a perfect bilateral symmetry, reflected by a bilaterally symmetrical arrangement of the resulting lateral clones. All descendent lineages show full bilateral symmetry, as is apparent from the equivalent lineage history of right and left counterpart clones (bottom panel). (B-C) For the bilateral founders in the dorso-medial (B) and ventro-medial (C) regions descending from 1 m-1121 sublineages, the lineage history of the left and right founder is very different. These founders originate at different branches of the quadrant homologue lineage tree and in some cases even differ in the lineage depth (light green, red, and dark green clones in panel B; light green clones in panel C). Two bilateral founder pairs - 1a-1121211 and 1a-1121121 (light and dark blue clone in panel C) and 1b-12111aa and 1b-121121b (dark green in D) originate from single quadrants. Note, that the cell divisions occurring at the lateral-most edge of this largely asymmetrical medial domain produce again symmetrical clones (sand and light brown clones in panel B). (D) The origin of A|C symmetry: The cells 1 m-12 divide spirally to produce accessory prototroch cells 1 m-122 and 1 m-1212. Subsequent cell divisions within 1c-12 and 1a-12 clone occur in a bilateral mode resulting in fully bilateral domains stemming from the A and C quadrant. [file 12915_2019_705_MOESM14_ESM.pdf]

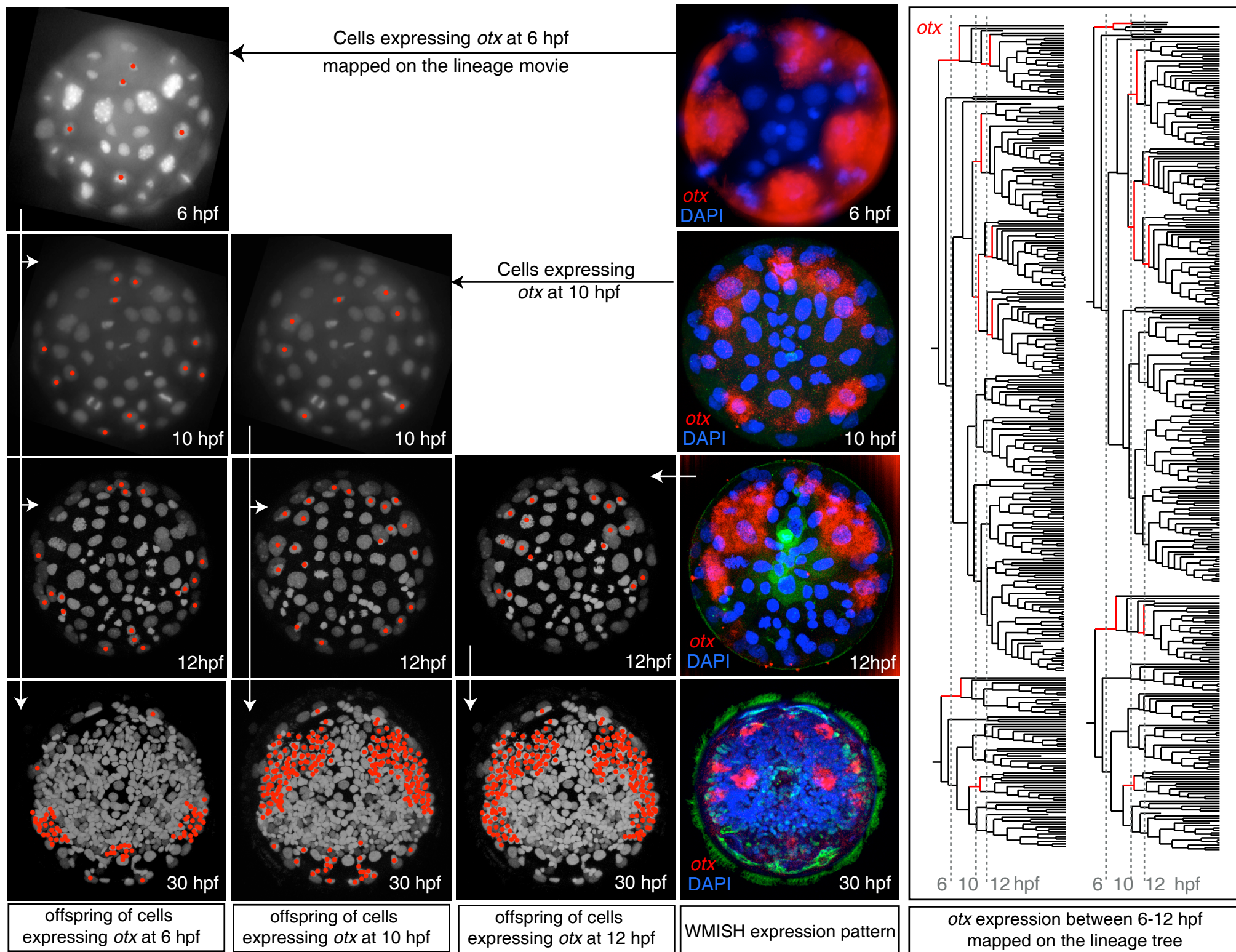

Supplement: Supplementary file 15 — Additional file 15: Figure S5. The dynamics of early otx expression. This figure shows the developmental expression of otx between 6 and 12 hpf mapped onto the cell lineage and reveals the dynamic switching of otx during episphere development. Otx expression visualized by WMISH (column next to the lineage trees) was mapped on lineage movie and lineage tree at indicated stages. Corresponding nuclei between the stained embryos and the movie frame (horizontal arrows) were identified manually at these stages. The vertical arrows indicate the theoretical clonal expansion of otx-positive cells at later stages. The comparison of such theoretical clonal expansion of otx-expressing cells at different stages to real expression pattern at a given stage (WMISH panel next to the lineage trees) shows that otx expression is not clonal and that the gene is dynamically switched on/off between cell cycles. The dynamic on/off switching of early otx expression is apparent in the lineage trees on the right-hand side, where the otx-expressing cells (in red) do not form a continuous lineage. [file 12915_2019_705_MOESM15_ESM.pdf]
